# Informing Efforts to Develop Nitroreductase for Amine Production

**DOI:** 10.3390/molecules23020211

**Published:** 2018-01-24

**Authors:** Anne-Frances Miller, Jonathan T. Park, Kyle L. Ferguson, Warintra Pitsawong, Andreas S. Bommarius

**Affiliations:** 1 Department of Chemistry, University of Kentucky, Lexington, KY 40506-0055, USA; 2 School of Chemical and Biomolecular Engineering, Georgia Institute of Technology, Atlanta, GA 30332-0100, USA; jpark5009@gmail.com; 3 School of Chemistry and Biochemistry, Georgia Institute of Technology, Atlanta, GA 30332-0100, USA; k.ferge@gmail.com; 4 Department of Chemistry, University of Kentucky, Lexington, KY 40506-0055, USA; warintra@brandeis.edu; 5 School of Chemical and Biomolecular Engineering, School of Chemistry and Biochemistry, Georgia Institute of Technology, Atlanta, GA 30332-0100, USA

**Keywords:** nitroreductase, flavoenzyme, enzyme-aided synthesis, structure-activity, structure-function, intertwined dimer, domain-swapped dimer, flavin

## Abstract

Nitroreductases (NRs) hold promise for converting nitroaromatics to aromatic amines. Nitroaromatic reduction rate increases with Hammett substituent constant for NRs from two different subgroups, confirming substrate identity as a key determinant of reactivity. Amine yields were low, but compounds yielding amines tend to have a large π system and electron withdrawing substituents. Therefore, we also assessed the prospects of varying the enzyme. Several different subgroups of NRs include members able to produce aromatic amines. Comparison of four NR subgroups shows that they provide contrasting substrate binding cavities with distinct constraints on substrate position relative to the flavin. The unique architecture of the NR dimer produces an enormous contact area which we propose provides the stabilization needed to offset the costs of insertion of the active sites between the monomers. Thus, we propose that the functional diversity included in the NR superfamily stems from the chemical versatility of the flavin cofactor in conjunction with a structure that permits tremendous active site variability. These complementary properties make NRs exceptionally promising enzymes for development for biocatalysis in prodrug activation and conversion of nitroaromatics to valuable aromatic amines. We provide a framework for identifying NRs and substrates with the greatest potential to advance.

## 1. Introduction

**Figure 1:**
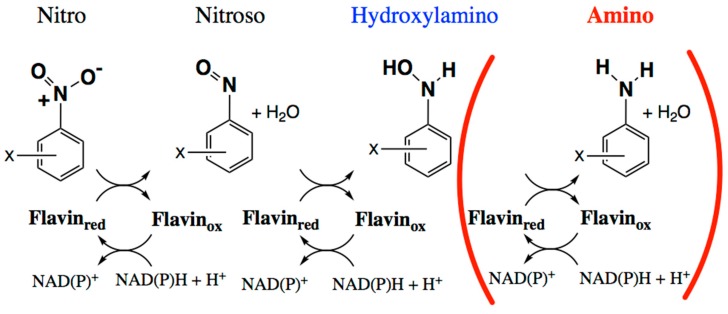
Biochemical reduction of nitroaromatic groups in sequential two-electron steps mediated by nitroreductase-related (NR-related) superfamily members [11,12,22]. The first two reductions appear general whereas the third appears more occasional and its occurrence is not as well understood [29].

Numerous efforts are underway to develop nitroreductase enzymes to activate prodrugs [1,2], remediate pollutants [3,4,5,6,7] and generate building-blocks for high-value pharmaceuticals [8]. However, reduction of nitrated aromatics has been a challenge to chemists since the original work by Haber in 1898 [9,10]. The yield of desired amines is commonly diminished by incomplete reduction to the nitroso and hydroxylamino products [11,12,13] (Figure 1). Moreover these partially-reduced compounds react with one-another to form additional by-products that can be toxic, and therefore require expensive purification steps (reviewed in [14]). Inorganic catalysts have shown promise but similarly produce byproducts and also must be quantitatively removed before the product can find pharmaceutical application [15]. Thus, there is a need for an efficient, clean method for producing aromatic amine precursors for use in pharmaceutical, animal health, or crop protection applications.

The use of enzymes in synthesis applications is gaining prominence [16,17,18,19,20,21]. NR is attractive because its broad substrate repertoire enables a single enzyme to transform a variety of substrates [11,17] and because it could provide a means of generating high-value aromatic amines from readily-available nitroaromatics [8]. 

The NRs of *Enterobacter cloacae* and close relatives have been shown to reduce nitrated aromatics through a series of two-electron steps via a ping-pong mechanism [11,12,22] (Figure 1). Previous work found that the well-characterized NRs from *Escherichia coli* and *E. cloacae* (*Ec*NfsB and *Ent*NfsB, respectively) reduce nitroaromatics to the corresponding hydroxylamines, not to the amines [11,12,22]. However, examples of aromatic amine production by members of this enzyme superfamily show that this can occur. Two clostridial NRs reduce TNT (trinitrotoluene) to 2-amino-4,6-dinitrotoluene [23], as have two from *Klebsiella* sp. C1 [24,25], a NR from *Bacillus cereus* [26], the so-called NfrA from *B. subtilis* [27] and more recently a NR from *Gluconobacter oxydans* 621H [28]. Therefore, we wish to learn what factors allow certain NRs to execute this chemistry and which substrates it is applicable to.

We proposed that insufficiency of driving force could provide a chemical explanation for nitro reduction only so far as the hydroxylamine [30], since the reduction midpoint potential (E°) for further reduction of aromatic hydroxylamines is considerably more negative, and thus less favorable, than the E°s of corresponding nitroso or nitro aromatics [31,32]. For nitrofurazone in an aqueous medium at pH 7.45, reduction to the corresponding hydroxylamine was observed at −270 mV (vs. NHE), but further reduction to the amine occurred at −830 mV [33]. For nitrobenzene only, the hydroxylamine was formed at pH 7.4, at −340 mV, however at pH 2.5 reduction to hydroxylamine occurred at −60 mV and further reduction to amine was observed near −600 mV [31]. Moreover the reduction mechanisms of nitroaromatics have proven to be complicated and dependent on the medium, proton sources and even the nature of the electrodes used [14,33]. In aprotic media, electrochemical and FTIR studies demonstrate that the mechanism of nitrobenzene reduction is dominated by two sequential one-electron (1-e) reductions to form nitroso, but that in protic media, sequential two-e reductions, to produce the nitroso and then the hydroxylamine, provide a better description [34,35,36]. In water, reduction of the nitroso intermediate is more facile than its formation [11,12,37], so electrochemical studies observe a single 4-e reduction of the nitroaromatic to the hydroxylamine, followed at lower E° by 2-e reduction to the amine, depending on pH [31,38]. Thus, we propose that an enzyme’s ability to provide protons to the reaction will play an important role in addition to the flavin’s E°. Nevertheless, diverse studies find that nitroaromatic compounds with higher reduction potentials are more rapidly reduced [37,39,40]. Therefore, we asked whether this trend might also apply to yield and product nature by testing the hypothesis that substrates with highly electron-withdrawing substituents would be more readily converted to the corresponding amines.

It is also possible that the structure of the enzyme’s active site determines whether a nitrated aromatic is completely reduced to the amine, or only to the hydroxylamine. There is considerable diversity among the active sites of so-called ‘nitroreductases’ because the name has been applied widely, including to enzymes whose function is now known to be an unrelated reaction [41,42,43,44]. A recent monumental effort documents relatedness among some 25,000 amino acid sequences attributed or related to NRs [41], confirming that the NR-related superfamily includes diverse enzymatic activities, but has been sparsely studied with regard to biochemical capabilities and physiological function. While the NR superfamily is comprised of at least 22 distinct major subgroups (Supplemental Figure S1), this paper focuses on just four, each named after a better-known biochemically-characterized representative that has been found to reduce nitroaromatics: NfsA (nitrofurazone sensitivity-A), NfsB, PnbA, and Frm2 (these names and HUB are those of Akiva, Copp et al. [41]). In what follows, we denote NR-related enzymes according to the subgroup to which they belong and use the source species to specify which variant is under discussion, for example *Ent*NfsB indicates the NfsB from *Enterobacter cloacae*. Considering that each of the subgroups can include enzymes with differing functions [45,46,47,48], we can expect to uncover more substrates and reactions as researchers undertake experimental studies of hitherto-uncharacterized subgroups and families within them.

Despite their prodigious diversity regarding reactions catalyzed, members of the NR-related superfamily share common core structure and almost all bind FMN or FAD either as cofactor or substrate [41] (FMN and FAD are flavin adenine mononucleotide and flavin adenine dinucleotide, respectively). Indeed, the chemical virtuosity of flavins is most likely a basis for the biochemical diversity of the superfamily. However, the shared core structure must also allow the different subgroups of the superfamily to promote different aspects of the flavin’s chemical repertoire. Thus it is anticipated that active site features shared within each subgroup interact with the flavin to modulate its activity in a common way [49,50,51], select a common category of substrate and/or position substrate in a way that is shared within the subgroup [52,53,54,55]. Conversely, given that some superfamily members have been reported to reduce nitroaromatics to corresponding amines, does this activity correlate with a particular subgroup, or active site features?

The PnbA-related enzyme from *Mycobacterium smegmatis* (*Ms*PnbA) has been shown to confer resistance to the anti-tuberculosis drug BTZ043 based on its ability to reduce this nitroaromatic drug to the corresponding amine [56]. Therefore, we have performed a basic biochemical analysis of this enzyme’s nitroreduction activity. We compared the *Ms*PnbA to the NfsB-related enzyme from *Salmonella typhimurium* (*St*NfsB). *St*NfsB differs by only eight out of 217 amino acids from the mechanistically-characterized *Ent*NfsB but has advantageous solution and stability properties [57]. Thus, our second hypothesis was that for a given compound, *Ms*PnbA would produce more of the amine product than *St*NfsB, and our experiments compared two different subgroups of the NR superfamily via these two enzymes.

We tested the hypotheses that (1) susceptibility of substrates to reduction would increase with electron-withdrawing substituents for both *Ms*PnbA and *St*NfsB; (2) *Ms*PnbA would produce more amine; and (3) *Ms*PnbA might also be expected to reduce the nitro substrates faster, if the product observed in previous studies [56] did not represent thermodynamic equilibrium. For substrates chosen to provide a spectrum of driving force for the reaction, our data demonstrate that a larger π system and more electron withdrawing substituents favor formation of amine product, but do not suffice. Nor did the *Ms*PnbA support more rapid nitro-reduction. However, our structure-based studies provide a path forward, confirming distinct placement of constraints on substrate binding relative to the flavin in different NR subgroups, and highlighting the unique architecture of NR superfamily members that appears to provide stabilization at a distance for its very versatile active site. Our work provides a rational basis for identifying promising NRs and substrates for screening. We suggest that the capacious active sites of members of the NfsA, Frm2 and primitive HUB NR subgroups will be good sources of enzymes for use in combination with substrates that have large aromatic π systems activated by electron withdrawing groups, for production of aromatic amines.

## 2. Results and Discussion

### 2.1. Amine Product Formation Is Low for Both Enzymes, but Large π Systems Seem Better

While trinitrotoluene (TNT) and some other nitroaromatics are documented to undergo reduction to the corresponding amines, many of the reports of the greatest conversion have employed intact bacteria or even consortia of multiple strains [29,58,59]. However, such systems provide limited control over the reaction outcome, and in several cases the enzyme responsible for amine formation has been shown to be other than a NR [60,61,62,63]. Moreover, the extent to which the amine accumulates depends on the ambient reduction potential [7,40,64]. Nevertheless, a route to formation remains a prerequisite for any accumulation.

To begin parsing the significance of substrate identity vs. enzyme identity we worked with purified enzymes and compared eight substrates, possessing a range of electron withdrawing groups and a range of aromatic π system sizes. We compared the members of two different NR subgroups: *St*NfsB and *Ms*PnbA. The NfsB from *S. typhimurium* has been studied extensively and is a close relative of the NfsBs from *E. cloacae* and *E. coli* for which detailed mechanistic information is available [12,17,22,50]. The PnbA from *M. smegmatis* converts the nitroaromatic drug BTZ043 to the corresponding amine with a yield on the order of 30% [56]. The relatedness of these two subgroups can be seen in Supplementary Figure S1.

**Figure 2:**
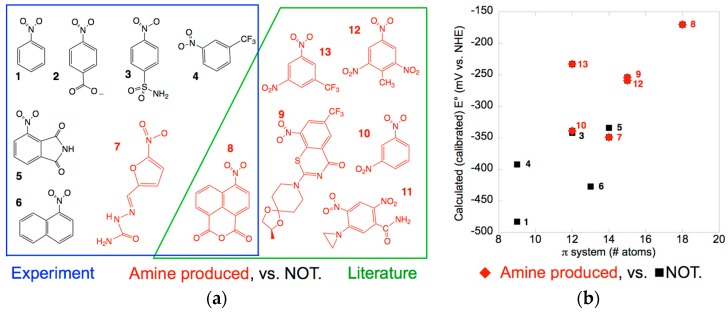
(**a**) Compounds investigated for amine formation by *Ms*PnbA and *St*NfsB. Compounds for which amine products were produced (**7**–**13**) are in red, those for which amine production was below our detection limit are in black (**1**–**6**). Compounds included based on literature reports are in a green box, compounds studied in this work are in a blue box. This figure provides the structures of BTZ043, **9** and CB1954, **11**; (**b**) Compound names and ChemSpider ID codes are given in Materials and Methods and Supplemental material.

Figure 2a shows the compounds tested, highlighting the two that produced significant amine product. Amine product was formed from compound **8** by both enzymes, however the amount (≈1%) was too low to support accurate quantitation. Similar results are reported for the NR from *Klebsiella* C1 which produced a 0.8% yield of 2-amino-4,6-dinitro toluene from TNT [25]. Compound **7** also produced amine but the absence of a standard prevented quantitation. This is a recurring challenge for environmental research where diverse products are formed but do not merit development of synthetic protocols (for another example see [26]). Thus, interpretation of the results must remain qualitative, but it is interesting that they were the same for the two enzymes. This is consistent with the similar midpoint potential of the *Ms*PnbA to that of *Ent*NfsB (−190 mV ± 30 mV). However, *Ms*PnbA did not yield significant amine product from 3-trifluoromethyl nitrobenzene **4** which reproduces the nitrated portion of BTZ043, although it converts BTZ043 **9** to the amine [56]. This suggests that the rest of the molecule plays a role in correctly positioning the nitro-derived hydroxylamine group for reduction by the enzyme. Indeed, the 4-nitro-1,8-naphthalic anhydride **8** that combines a larger aromatic π system with electron withdrawing substituents underwent reduction to the amine consistent with literature reports [59]. Additionally, our findings document amine production from a member of the NfsB subgroup.

The structures of the substrates assayed, as well as those of known amine-producers BTZ043, TNT and the chemotherapeutic agent CB1954 [23,24,25,26,28] suggest that electron withdrawing groups and/or a large π system favor progression to the amine product (Figure 2a). Indeed, the literature documents robust correlations between the E° and rate of biotic (whole cells) and abiotic nitrogroup reduction for nitroaromatics [39,40]. Experimental values are only available for a few of the compounds of interest, but high-level computation has demonstrated methods for achieving accurate computed values of E° [65,66]. Our more pragmatic goal is to test the hypothesis that amine production is enhanced by electron withdrawing substituents, so we need a good description of the trend but not accurate individual values [67]. We found that this was provided by a medium-sized basis set in the gas-phase calculations (Supplemental Figure S2, Table S1). We refer to our semi-empirical values as *calibrated calculated values* (E°_c_) to distinguish them from measurements or fully *ab-initio* computations. However, these less demanding calculations provide a numerical index for extent of electron withdrawal (stabilization) from the π system and thereby make it possible to treat the more complicated molecules in our set.

Detailed studies have also explored dependence on hydrophobicity of substituents and have shown that these can play important roles in quality of productive binding [68,69,70]. This approach is complicated in the case of nitroaromatics because they are so electron withdrawing that they alter the polarity of the other substituents [71]. However, the significant differences between the two enzymes we are comparing allow that even very simple contributions to productive binding can provide insight, so we considered total substrate volume, log(P) (P is the octanol/water partition coefficient), and extent of the π system, because crystal structures indicate that substrates bind via π stacking against the flavin. 

The distribution of amine-producing substrates in a space of calibrated calculated E°_c_ vs. size of the π system suggests that both factors contribute to the likelihood of amine formation (Figure 2b). It is not surprising that the calculated E°_c_s trend with the extent of the π system, as a larger π system better delocalizes additional charge and thereby favors reduction (raises E°). An analogous plotagainst calculated total volume of the molecules reveals a qualitatively similar though slightly less separated distribution of compounds that yield amine (Supplemental Figure S3a) whereas a plot against log(P) indicates that low polarity is less important (Supplemental Figure S3b), compare with [2]. Future studies should measure K_d_s and K_M_s, however the two simple measures we describe here already suggest that smaller nitroaromatics will be less likely to undergo full reduction and therefore that the most useful enzymes will be those able to accommodate larger substrates. This is the first correlation of which we are aware between amine formation and molecular properties of the parent nitro compounds.

### 2.2. Electron Withdrawing Groups Favour Reduction of Nitro Substrates

**Figure 3:**
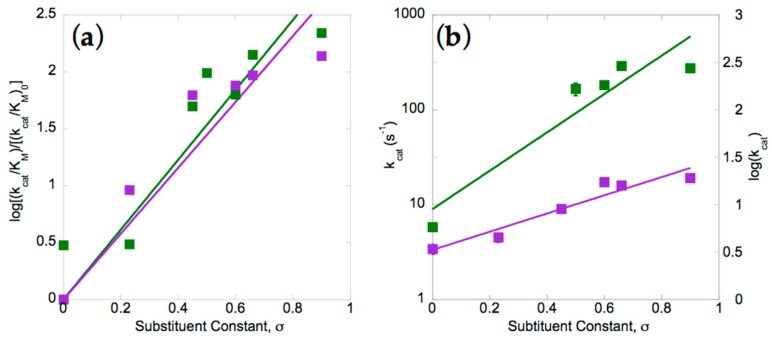
Dependence of enzymatic reaction rates on *para* Hammett constant, for *St*NfsB (green) and *Ms*PnbA (purple) [77]. (**a**) The second-order rate constant (k_cat_/K_M_) for each substituted substrate was divided by the value obtained for (unsubstituted) nitrobenzene before taking the log to get log[(k_cat_/K_M_)/(k_cat_/K_M_)_0_], which was plotted against the Hammett para substituent constant σ. Log[(k_cat_/K_M_)/(k_ca_t/K_M_)_0_] plots yielded ρ values of 3.1 ± 0.2 (*St*NfsB, R^2^ = 0.86) and 2.9 ± 0.2 (*Ms*PnbA, R^2^ = 0.83); (**b**) the first-order rate constants are plotted without normalization, yielding for *St*NfsB a slope of 2.4 ± 0.4 and an intercept of 0.9 ± 0.3 (R^2^ = 0.88), and for *Ms*PnbA a slope of 1.0 ± 0.1 and intercept of 0.5 ± 0.08 (R^2^ = 0.92). See Methods for the equation and also Supplemental Table S2 and Figure S4 for compounds, and details.

Our amine yields were too low to provide quantitative distinctions between different substrates. However, when initial velocity of nitro group reduction was compared, rates varied by over two orders of magnitude and therefore provided considerably better discrimination. Numerous studies have demonstrated that rates of reduction of nitroaromatics increase with electron withdrawing substitution on the ring, hence we compared the two enzymes with respect to their Hammett plots [28,37,39,40,72]. Plots of the log of the second-order rate constant (k_cat_/K_M_) were produced after dividing by the rate measured for the unsubstituted parent compound: nitrobenzene. Figure 3a shows that the two enzymes have similar reaction constants of ρ = 3.1 ± 0.2 and 2.9 ± 0.2 for *St*NfsB and *Ms*PnbA, respectively. The positive values for the reaction constant are indicative of accumulation of negative charge in the transition state of the rate-limiting step [73,74], consistent with the mechanism determined for *Ent*NfsB involving hydride attack on the nitro of *p*-nitrobenzoic acid [22,75]. The magnitude greater than one indicates that the reaction is more sensitive than is deprotonation of carboxyl functionality [73,74,76].

Although the two enzymes appear similar based on reaction constants, comparison of the dependencies of their first-order rate constants reveals a much smaller sensitivity electron withdrawing groups in the case of *Ms*PnbA than in *St*NfsB (slopes of 2.0 and 1.0, respectively, Figure 3b). The rate constants are simply lower for *Ms*PnbA, so it may be that additional components of the reaction are rate-contributing in *Ms*PnbA but too fast to be rate-contributing in *St*NfsB. The similar overall dependencies of the two enzymes at low substrate concentrations (Figure 3a) thus appear to mask compensating differences in the rate-contributing steps. In summary, our data confirm other studies that support the choice of substrate as very significant to the rate of nitroaromatic reduction, but add that different members of the NR superfamily appear to achieve their catalytic rate enhancements via different rate-limiting steps.

### 2.3. Differences between Subgroups in the NR Superfamily That Could Affect Nitroreduction Activity

To augment the substrate’s intrinsic propensity for reduction the enzyme must engage it in interactions that stabilize the transition state and position it for reaction by the reduced flavin (and a proton donor). However, recent studies on *Ent*NfsB demonstrate that substrate appears to bind in a poor geometry for nitroreduction [50]. Thus, a crucial second avenue for improvement is identification of active sites that provide geometry more conducive to reaction. For this, we can exploit the diversity of the NR superfamily.

Even before the landmark study of Akiva and Copp [41], it was evident that *Ms*PnbA provided a substrate binding context distinct from that of the NfsB family that has been so extensively studied [56,78]. This was captured by the Akiva/Copp framework which groups the *M. smegmatis* enzyme as a member of the PnbA subgroup but the *S. typhimurium*, *E. coli* and *E. cloacae* enzymes in the NfsB subgroup (Supplemental Figure S1). Our structure-based comparisons identified NfsA from *E. coli*, *Vibrio harveyi, B. subtilis* and several other organisms as a third category, coincident with the NfsA subgroup [41], based on their shared C-terminal extensions. Akiva and Copp identified the so-called ‘HUB’ subgroup of potentially primitive NRs lacking the structural features characteristic of the NfsB, PnbA or NfsA subgroups [41] as well as subgroups uniting more specialized members of the superfamily that catalyze reactions very different from nitroreduction, such as BluB and Iyd [41].

**Figure 4:**
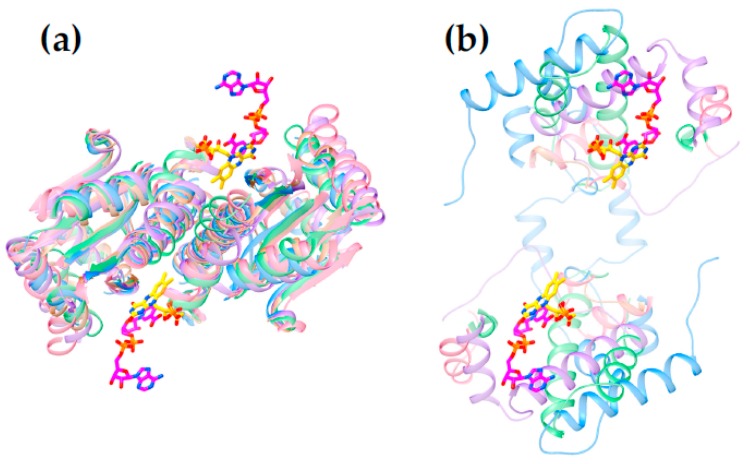
Relationship of flavin position and NADH binding site of NfsB relative to core structure (**a**) and distinguishing structure (**b**). Ribbon structure characteristic of the NfsA subgroup is in blue, that of NfsB is in green, that of PnbA is in purple and that of Frm2 is in pink. Structures used to represent these subgroups are 1F5V (NfsA), 5J8D (NfsB), 2WZW (PnbA) 2IFA (Frm2) and 3E35 (HUB, rust). Flavin and NADH position numbers are provided in Supplemental Figure S6.

**Figure 5:**
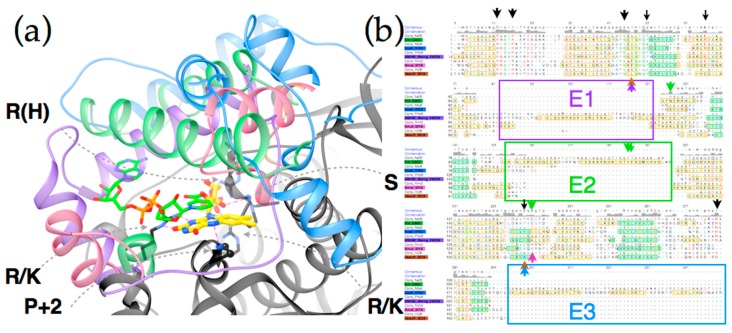
Comparison of the different substrate binding cavities, and their different origins in the sequences of PnbA (purple), NfsB (green), NfsA (blue) and Frm2 (pink). Panel (**a**) shows the contrast between exposed flavin binding pocket provided by conserved core structure (black) vs. enclosed substrate binding cavities formed by the distinguishing structures of the different subgroups. A map of amino acid conservation onto backbone structure for each of the current five subgroups is provided as Supplementary Figure S8. Core structure interactions that appear to stabilize FMN binding are indicated by grey dashed arrows. R(H) means Arg but sometimes His, R/K means Arg or Lys, P + 2 means that the second residue after a Pro contributes the interaction, S means Ser. The FMN from NfsB is included in yellow and an NADH analog bound to the NfsB model is in green, both with non-C atoms coloured by atom [50]; (**b**) Shows an alignment of consensus and representative sequences from each of these subgroups and HUB (brown label). The three insertions/extension in the sequence giving rise to the distinguishing structure are in boxes colored according to the subgroup in which it is best developed and labeled ‘E1’, ‘E2’ and ‘E3’ as per Akiva, Copp et al [41]. Yellow shading denotes alpha helices and green denotes beta strands. Black arrows indicate the locations of core residues interacting with the FMN, and arrows colored according to the above subgroup identify residues mentioned in the text as possibly constraining substrate binding and/or modulating flavin activity. A larger multi-sequence alignment is provided as Supplemental Figure S9 and those of the subgroups are available via the Structure Function Linkage Database [48].

The enzymes so far reported to convert aromatic nitro groups to the corresponding amines occur in the subgroups NfsA (four exemplars), NfsB and the closely allied MhqN (two exemplars), TstD (one), PnbA (one) and HUB (one). Thus, it is already clear that this activity is not unique to a particular subgroup. Considering the extent to which the few documented cases are dispersed, it is likely that additional subgroups will be found to possess this ability. Therefore, the choice of specific subgroup to investigate can in principle be made on the basis of compatibility with substrates of interest, for example those with an extended π system and electron withdrawing groups. To this end, we compared structures of the subgroups NfsB, PnbA, NfsA and HUB (all include an amine-producing enzyme) with the Frm2 subgroup which includes many superfamily members from yeast [41] and provides a particularly open active site (see Figure 4, Figure 5 and Figure S7).

### 2.4. Active Site Constraints on Substrate Binding Mode and Orientation

The available structures of NR superfamily members display common themes, some shared but others specific to subgroups. Several of the different subgroups identified by the larger sequence-based analysis of Akiva and Copp [41] also emerged on the basis of structural motifs (Supplemental Figure S1 vs. S5) and correspond to the subgroups NfsA, NfsB, PnbA and Frm2. The NRs classified in the HUB subgroup are also of interest for enzyme engineering because they were found to be the least specialized [41].

**Figure 6:**
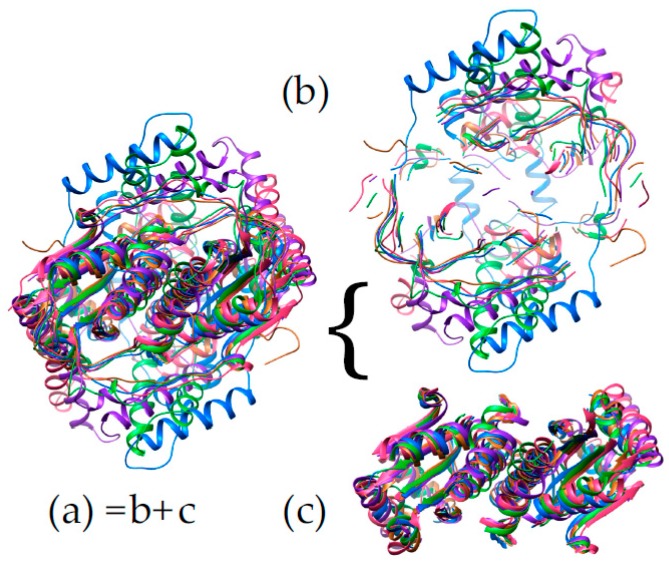
Superposition of NR-related superfamily members. (**a**) Common core structure is evident in an overlay of representatives of each of the NfsB (green), NfsA (blue), PnbA (purple) Frm2 (pink) and HUB (rust) subgroups. Panel (**c**) extracts the core secondary structure shared by the different subgroups (same overlay), while (**b**) shows all backbone ribbon present in (**a**) but excluded from (**c**). Structures used are 1F5V (NfsA), 5J8D (NfsB), 2WZW (PnbA), 2IFA (Frm2) and 3E39 (HUB). All molecular graphics were produced using Chimera [79].

Figure 6 reveals that the structures possess a core common to the different subgroups (Panel 6c). Two domains are seen, related by a C2 axis pointing out of the page as shown, consistent with the dimeric nature of all but just a few superfamily members (in which cases a gene duplication and fusion preserves the two-domain structure in a single, doubly-long peptide [41]). Additional secondary structure and strands on the periphery are shared within subgroups but differ between them (“distinguishing structure”, Figure 6b). The distinguishing structural elements are not randomly distributed, but rather concentrated around the sites of flavin and substrate binding (Figure 4), where they appear to be ideally positioned to constrain the substrate-binding mode or impose selectivity. For example the distinguishing structure of PnbA excludes NADH as the reducing substrate in the binding mode used by NfsB, because a helix that characterizes the PnbA subgroup occupies the space employed to bind the NADH adenine ring by NfsB subgroup members (Figure 5a, NADH is reduced nicotinamide adenine dinucleotide, Supplemental Figure S6). The distinguishing structures of the different subgroups shown in Figure 4b are clearly distinct from one-another yet occupy essentially the same space.

Thus, we view the different subgroups as opportunities to differently orient the substrate relative to the flavin and thus direct reactivity at different positions of a substrate, or to select different substrates altogether. In particular, we propose that NfsB’s poor geometry for reduction of nitrogroups is unlikely to be general, and that another subgroup may provide a more suitable geometry for its reduction to amine. Moreover, given nitroaromatic binding in *Ent*NfsB treats the nitroaromatic like an analog of NADH, placing the nitrogroup where NADH’s amide binds, and the non-target aromatic ring near N5 where NADH’s hydride-bearing C4 binds [50], we propose that a subgroup that does *not* employ NADH as the reducing substrate may be the best choice of platform for aromatic nitro group reduction. The distinguishing structure of the different subgroups constrains the substrate-binding cavity on different sides (Figure 5a and Figure S7) consistent with the provenance of this structure from different locations in the amino acid sequence (Figure 5b [41]). Moreover some subgroups simply do not constrain the substrate binding site as tightly, for example solved structures from Frm2 and HUB afford more open active sites than PnbA (Supplemental Figure S7).

Figure 5 shows the complementary roles played by conserved core structure (black) and distinguishing structure (four colors). Key requirements for FMN binding are met by conserved amino acids in the core sequence, including an Arg (or His) that provides electrostatic stabilization of FMN’s phosphate, another Arg (or Lys), and a backbone oxygen that hydrogen bond with ribose OH groups (the residue contributing the backbone O is generally conserved as Ser, the other ribose hydroxide points towards solvent). A third Arg (or Lys) is positioned such as to stabilize negative charge in the N1-O2 region of the reduced flavin (Supplemental Figure S6 for numbering). Finally a hydrogen bond from backbone NH to flavin N5 is provided by a residue that is conserved as small and constitutes a juncture between a central beta strand and a conserved alpha helix (the second residue after a conserved Pro under the *si* face of the flavin, as drawn). Thus, core side chains and structure satisfy the requirements for binding polar and charged functionalities of FMN. 

The conserved core also displays some subgroup-specific residue identities that are expected to alter reactivity and substrate preferences. A tight turn is conserved above the *re*-face of the flavin as drawn (Figure 5a), but different subgroups differently constrain the volume available for substrate binding via the size of the side chain present here. For example, many HUB subgroup members place a Lys here whereas many PnbA members possess a Cys. The *si* face of the flavin is effectively blocked in the subgroups discussed here, but a Pro is used by NfsB and PnbA whereas Trp is present in the HUB subgroup members and Tyr is used by NfsA allies [7,80]. Similarly, interactions contributed by distinguishing structures likely underlie distinct reactivities displayed by the different subgroups [41]. For example NfsB and PnbA provide bidentate H-bonding to the flavin N3H and O4 from a conserved Asn side chain and in NfsB a conserved Glu organizes water near the flavin N5, but this is a His in Frm2 [80]. The E2 excursion of NfsB walls off the substrate-binding cavity from bulk solvent with a pair of aromatic side chains (Tyr123 and Phe124 in *Ent*NfsB). These are proposed to apply soft selection on what can bind [80]. The analogous residues in *Ec*NfsB affect the enzyme’s activity [7] and modulate its regioselectivity [53,54]. Changes to Ser41 in the tight turn above the flavin *re* face (see above) as well as substitution of Arg225 and often Phe227 in the distinguishing structure that encloses the substrate binding site of *Ec*NfsA emerged upon directed evolution for activation of the prodrug PR-104A and were found to improve the capacity of the substrate binding site [2]. Thus, we argue that the large distinctions between the structures and sequences that enclose the active site predict that different subgroups will share different reactivities [81] and therefore that subgroups with demonstrated capacity to bind substrates with a large π system offer better prospects for discovery or engineering of amine-producing enzymes.

### 2.5. ‘Intertwining’ of the Two Peptide Chains May Enable the NR Dimer to Tolerate Diverse Substrates and Diverse Interactions in Its Active Site

**Figure 7:**
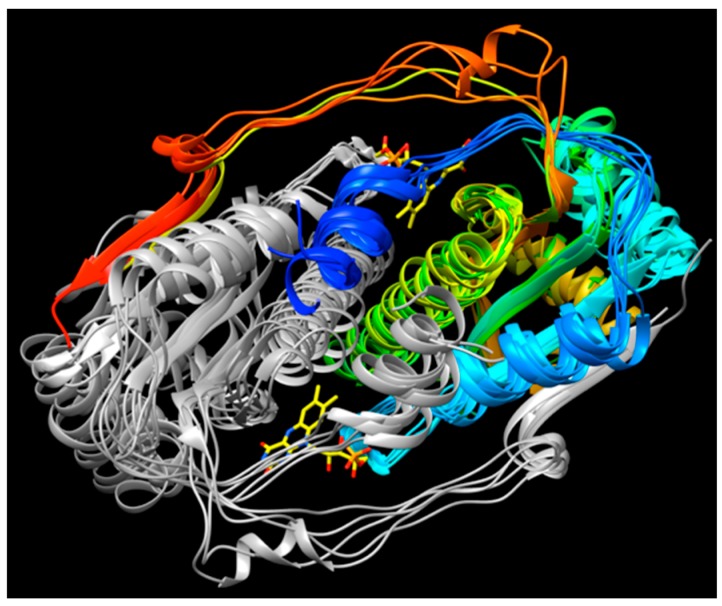
‘Intertwining’, between domains of both the N and the C termini of the two chains. The A chain is colored according to a rainbow progression from blue at the N-terminus to red at the C-terminus; the B chain is in grey. The N-terminal helix of chain A nestles amid helices of chain B and C-terminal residues from chain A contribute a strand (red) to the beta sheet of domain B (left).

Binding of both substrate and flavin occur in the interface between monomers, for the NR superfamily members for which structures have been solved. This might be imagined to pry the two domains apart and weaken the dimer. We propose that the stability needed to permit ligand binding between monomers is provided by NR’s conservation of two intertwined structural motifs. First, the N-terminal helix from one chain nestles between two helices of the other monomer (blue helix, centre of Figure 7). Second, the C-terminus of one chain contributes the fifth strand of a beta sheet central to the other monomer (red at 9:30 o’clock in Figure 7).

**Figure 8:**
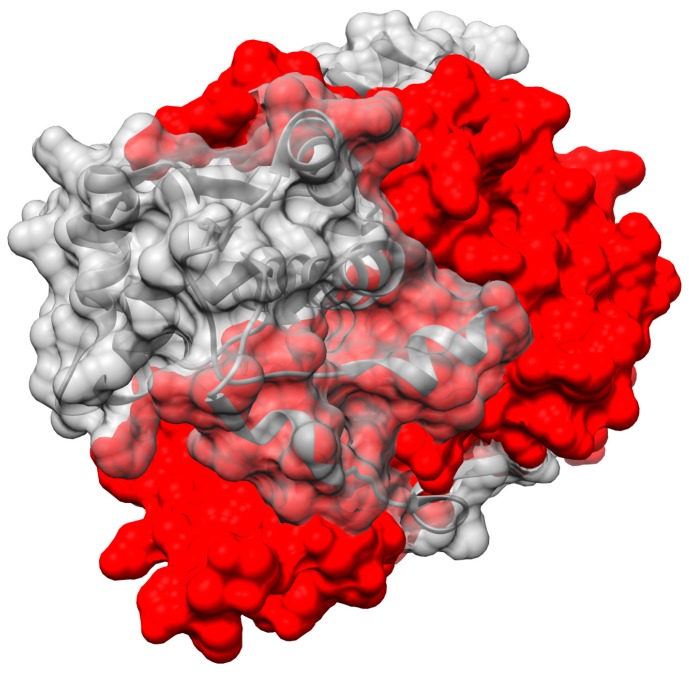
The extensive buried surface area of NfsB dimer (that of NfsA is larger still). The A chain is colored red with an opaque surface; the B chain is in grey with a partially transparent surface. One monomer wraps extensively around the other and buries much of its surface.

‘Intertwining’ among units of oligomers has been described in numerous other systems as increasing the stability of the quaternary structure [82,83]. Indeed, the dimeric structure the NR-related superfamily features a very large contact area between monomers, considering the monomer’ modest size (Figure 8). This is especially striking for the NfsA subgroup where the C-terminal excursion wraps around the other monomer thereby burying some 5500 ± 300 A^2^. PnbA and NfsB members have smaller buried interfaces (5100, 4600 Å^2^) and the Frm2 and HUB subgroups bury the least with 3900 and 4200 ± 100 Å^2^, respectively (Supplemental Table S3.) However, even these values are enormous in the context of protein dimers in general. Thornton’s team analyzed 76 homodimers and found only six with contact areas greater than 4000 Å^2^ (the average was ≈ 1600 Å^2^) [84]. Values greater than 2000 Å^2^ are considered large [82]. Comparing with a similar-sized dimer with its active sites at the dimer interface, one monomer of Mn-containing superoxide dismutase has a surface area of 9300 Å^2^ and dimerization buries 12% whereas the *Ent*NfsB monomer has a surface area of 13,200 Å^2^ and dimerization buries 35%. 

The large contact area between monomers is conserved across all the NR superfamily members for which multiple structures have been deposited, and could be an important contributor to the high stability of the dimer [82,85,86]. This would be essential in order for the dimer to accommodate the flavin and the substrates in the interface without dissociating. We propose that NR’s doubly-intertwined dimer motif is related to flavin and substrate activation as well, because the extensive inter-subunit interactions can provide substantial stabilization of the dimer to offset energetic costs of stabilizing reaction transition states.

NR’s architecture may also be crucial to the superfamily’s ability to support varied chemistry. In NR’s structure, amino acid changes associated with different reactivity can be peripheral to individual domains rather than internal, yet still point into the active site located between monomers. Additional determinants of flavin reactivity and substrate selectivity are located in the distinguishing structures of the different subgroups that are also peripheral rather than integral to the core. Meanwhile the active site is stabilized by interactions located far away in both sequence and space, thus imbuing this superfamily with great tolerance to variations within the active site. Just as the prodigious chemical variety represented in this superfamily can be traced to the large chemical repertoire of the cofactor, a flavin, we propose that stabilization of the dimer (and thus the active site) *from a distance* is a component in the evolutionary success and versatility of this fold. We speculate that different variants of the theme have been able to evolve to emphasize different elements of flavin chemistry and different substrates precisely because the structure is inherently tolerant of changes to the active site, stabilized by intertwined structural motifs and enclosed by excursions conserved within subgroups but external to the conserved core structure.

## 3. Materials and Methods 

### 3.1. Materials

NADH and NADPH were obtained from Amresco (Solon, OH, USA) and EMD chemicals (Gibbstown, NJ, USA), respectively. All other chemicals such as substituted nitro compounds and salts used for buffers were obtained from Sigma–Aldrich (St. Louis, MO, USA).

### 3.2. Genes

*nfnB* from *Mycobacterium smegmatis* was extended at the C-terminus to produce a poly-His tag and as such was the generous gift from Dr. Giovanna Riccardi at the Università degli Studi di Pavia [56]. The gene *stnr* from *Salmonella typhimurium* [87] was used as in ref. [57].

### 3.3. Protein Expression and Purification

The genes *stnr* and *nfnB* (coding for *St*NfsB and *Ms*PnbA) were expressed in pET-28a vectors [88]. Plasmids were transformed into BL21 (DE3) cells for expression. A number of 1 L cultures were inoculated using 1% (*v*/*v*) overnight precultures with 30 µg/mL of kanamycin, and grown at 37 °C. When the optical density at 600 nm (OD_600_) reached 0.5~0.7, protein expression was induced with 1 mM isopropyl β-d-1-thiogalactopyranoside (IPTG). After induction, cultures for *St*NfsB were grown at 37 °C for 8 h, and *Ms*PnbA was expressed overnight (~16 h) at 28 °C, which was found to improve yields and quality of enzyme. Cells were harvested by centrifugation at 4050× *g* for 10 min in a Beckman preparative centrifuge. Pellets were either directly used for purification or stored at −80 °C.

*St*NfsB was purified according to a previous study with modifications [57]. Purification was performed at 4 °C or in an ice bath, and centrifugation steps were carried out with a Sorvall RC5Bplus centrifuge at 26,940× *g* for 30 min. Cell pellets were suspended in 20 mL of 50 mM Tris-Cl pH 7.5 (buffer A) and sonicated at 14 Watts for 30 s nine times with 30 s breaks in between. Lysed cells were centrifuged, and saturated ammonium sulfate solution was added to the clarified lysate to a final concentration of 40%. The sample was equilibrated with constant mixing for an hour and then centrifuged. Additional saturated ammonium sulfate solution was added to the supernatant to a final concentration of 70%. After one hour of equilibration the sample was centrifuged, and the pellet was resuspended with 5 mL of buffer A. Next, the sample was dialyzed twice against 500 mL of buffer A for two hours each time. The protein was filtered through 0.8 and 0.2 µm membrane microfilters in series. Filtered protein solution was injected onto an ÄKTAexplorer™ (GE Healthcare Life Sciences; Piscataway, NJ, USA) equipped with a HiPrep 16/10 DEAE anionic exchange column, pre-equilibrated with buffer A. Gradient separation was performed from 10% to 30% using buffer A with 1 M NaCl (buffer B) over 15 column volumes (CVs). Fractions were collected, assayed for activity, and concentrated to less than 2 mL with 3 kDa molecular weight cutoff (MWCO) Nanosep^®^ centrifugal devices (Pall; Port Washington, NY, USA). FMN was added to a final concentration of 0.2 mM. After incubation for one hour the enzyme was purified from excess FMN on a HiPrep 16/60 Sephacryl S-300 column developed with an isocratic flow of buffer A with 150 mM NaCl. 

Purification of *Ms*PnbA exploited the his-tag and affinity chromatography using Ni-nitrilo triacetate resin. Cell pellets were resuspended in 20 mL of 50 mM sodium phosphate, 300 mM NaCl, and 20 mM imidazole at pH 8.0. Cell slurry was sonicated at 14 Watts for 30 s nine times with 30 s breaks in between. Lysed cells were centrifuged, and the resulting supernatant was incubated with FMN at a final concentration of 0.2 mM for one hour. The sample was loaded onto 2 mL of Ni-NTA resin with gentle rocking at 4 °C for 45 min. The resin was washed with five column volumes (CVs) of 50 mM sodium phosphate (pH 8.0), 300 mM NaCl, and 50 mM imidazole two times. The protein was then eluted with 0.5 CVs of 50 mM sodium phosphate (pH 8.0), 300 mM NaCl and 250 mM imidazole eight times.

Sodium dodecyl sulfate polyacrylamide gel electrophoresis (SDS-PAGE) was used to identify fractions with the highest purity of protein. Active fractions were identified and stored at −20 °C with 50% (*v*/*v*) glycerol.

### 3.4. Measurement of Enzyme Kinetics

Initial specific activity measurements were conducted on a DU 800 spectrophotometer (Beckman Coulter; Brea, CA, USA). Studies were performed with 0.1 μM enzyme, 0.5 mM NADH, and 0.1 mM nitro substrate at 25 °C. Oxidation of the co-substrate NADH was monitored at 370 nm (ε_370_ = 2660 M^−1^·cm^−1^ [89]) as a function of time to determine the initial reaction velocity.

To determine the apparent Michaelis-Menten kinetic parameters, *k_cat_* and the *K_M_* for NAD(P)H ($K_{M}^{NAD\left(P\right)H}$) 2,4-dinitrotoluene (2,4-DNT) was used as a model substrate. Concentrations ranging from 0.013 to 0.5 mM and 0.05 to 2 mM were used for NADH and NADPH, respectively. When studying *St*NfsB, a range from 12.5 μM to 12 mM was studied for each substituted nitro compound depending on its solubility, with 0.5 mM NADH. Prior to addition of the nitro-substrate, a baseline was collected to permit calculation of uncoupled NAD(P)H oxidation. For *Ms*PnbA, a microtiter plate (MTP) assay was used with a Synergy H4 Multi-Mode Plate Reader (BioTek; Winooski, VT, USA) in 96-well plates. Substrate concentrations ranging from 0.01 mM to 4 mM were studied to measure the kinetic parameters.

Data were fit with OriginPro (v 9.0.0) software (OriginLab; Northampton, MA, USA). Kinetic data for NAD(P)H and nitro substrates were fit according to the Michaelis-Menten equation (Equation (1)) where one substrate was varied while the other was fixed [90], and where *v* is the reaction rate, [*E*]_0_ is the enzyme concentration and [*S*] is the varied substrate concentration.

$$v=\frac{k_{cat}{\left[E\right]}_{0}\left[S\right]}{K_{M}^{S}+\left[S\right]}.$$

The rate constants obtained, *k_cat_* and/or *k_cat_*/$K_{M}^{S}$ re measured for a series of substrates varying with respect to a *para* substituent. To assess the extent to which electron withdrawing substituents stabilize the transition state we analyzed the results according to the Hammett Equation (2) in which *Y* is the rate (*k_cat_* or *k_cat_*/$K_{M}^{S}$)the subscript *i* indicates either substrate ‘*I*’ and subscript 0 indicates the unsubstituted parent compound, *σ* is the *para* Hammett substituent constant (Table S2) and *ρ* is the reaction constant obtained from a linear fit of log (*Y_i_*/*Y*_0_) vs. *σ* [73,74].

$$log\;\left(\frac{Y_{i}}{Y_{0}}\right)\;=\;\sigma \rho .$$

Although the plots display considerable scatter, additional constants such as the π constant for hydrophobicity are not considered applicable to nitroaromatics [71]. The dependences obtained based on σ alone were nevertheless adequate for the objective of comparing the behaviours of the two enzymes.

### 3.5. Detection of Amine Products

The products of reduction of diverse nitroaromatic substrates were screened for amine formation using HPLC (high-performance liquid chromatography) and mass spectrometry (MS). The reactions each contained 100 μM substrate and 1 μM NR in 50 mM Tris-Cl buffer (pH 7.5). A continuous supply of reduced NADH was ensured using 5 U/mL glucose dehydrogenase (GDH), 10 μM NAD^+^ and 10 mM dextrose. Products were extracted with 0.5 mL of ethyl acetate and either analyzed by high-performance liquid chromatography (HPLC) or LC-MS (liquid chromatography- mass spectrometry) after drying under a gentle stream of argon gas and resuspension in water:acetonitrile (50:50). The enzymatic reactions and extractions were carried out in an anaerobic bag. All reactions with ensuing analyses were performed at least twice.

HPLC was conducted with a Shimadzu LC-20AT pump, Phenomenex Luna^®^ 5 µM C18 100 Å 250 × 4.6 mm (Torrance, CA, USA), and SPD-M20A prominence diode array detector (PDA). Separation was achieved with an isocratic flow of water:acetonitrile in ratios that were optimized for individual substrates (see Table 1). LC-MS analysis was performed with an Agilent 6320 Ion Trap LC-MS system (Santa Clara, CA, USA) with Electrospray Ionization (ESI). Separation was achieved on an Agilent ZORBAX SB-C18 5 μM 150 × 0.5 mm capillary HPLC column with an isocratic flow of water:acetonitrile (50:50) at a flow rate of 0.1 mL/min. The different properties of the compounds compared made it essential to quantify each amine product at its λ_max_ and sometimes a secondary wavelength to differentiate from other byproducts as described in Table 1. We judge that we were able to detect amine whenever it was formed at >1%, and that failure to detect amine indicates that yield was <0.5%, on average. Product identities were confirmed by MS including analysis of fragmentation products.

### 3.6. Measurement of Enzyme Reduction Potential

The two-electron reduction potential of *Ms*PnbA was evaluated by reducing it in the presence of phenosafranine as the reference dye (18 µM) according to the method of Massey [91]. Reducing equivalents were provided using the xanthine and xanthine oxidase system (400 μM, 3 nM, respectively) and the titration proceeded overnight without intervention but with 2 μM of the mediator benzyl viologen present to ensure equilibrium throughout. During the reduction process, absorbance at 454 and 521 nm was measured at intervals. This was then used to calculate the amounts of oxidized *Ms*PnbA and dye present, over the course of the reduction. A length of 521 nm was used to evaluate the concentration of oxidized dye (ε_521_ = 44.7 mM^−1^·cm^−1^) and its extinction coefficient at 454 nm (ε_454_ = 9.85 mM^−1^·cm^−1^) was used to correct for a small contribution at that wavelength, which was then used to calculate the % of *Ms*PnbA in the oxidized state. A plot of log(E_red_/E_ox_) vs. log(D_red_/D_ox_) displayed a linear correlation between the two with a slope of 1 confirming applicability of the Nernst equation (Equation. 3) where *E*_ox_ is the concentration of oxidized *Ms*PnbA and *E*_red_ is the concentration of reduced *Ms*PnbA (= total concentration−oxidized concentration), *D*_ox_ and *D*_red_ are similarly defined for the dye, *n_d_* and *n_e_* are the number of electrons taken up by the dye and the enzyme, respectively (=2). The intercept was used to calculate the midpoint potential of the *Ms*PnbA enzyme $E_{e}^{o}$()f −190 ± 30 mV, using the known reduction midpoint potential of the dye $E_{d}^{o}$( −252 mV [92]).

$$log\left(E_{red}/E_{ox}\right)=\frac{n_{e}}{n_{d}}log\left(D_{red}/D_{ox}\right)+\frac{n_{e}}{0.0592}\left(E_{e}^{0}-E_{d}^{0}\right).$$

### 3.7. Computations

The method developed employs relatively low level of theory and gas-phase treatment in order to accommodate the relatively large molecules included, without recourse to supercomputers or software that could be inaccessible to general practitioners. All computations were implemented in Spartan’16 (WaveFunction) [93]. All molecules with multiple conformations were first treated via a molecular mechanics force field Monte-Carlo search for minimum-energy conformations and the conformations representing 5% or more of the population were geometry optimized using ωB97X-D/6-31G* *in vacuo* [94]. Only the lowest-energy conformation was retained. The alkyl amine substituent of BTZ043 (NC_5_H_8_O_2_C_3_H_6_) was truncated by replacement with simple amine (NH_2_) for the purposes of thermodynamic properties only. All structures were geometry optimized further using ωB97X-D/6-311+G** and then used to calculate vibrational modes, thermodynamic quantities and molecular properties [93]. The choice of basis set size was validated by trial calculations using increasing sizes of basis sets that demonstrated convergence of energies, as well as comparisons of methodologies for calculation of reduction midpoint potentials [34,65,66].

All potentials were calculated relative to that of nitrobenzene (BzNO_2_). By computing the free energies of the individual oxidized and reduced species we obtained the change in free energy ΔG°_rxn_ for the reaction ArNO_2_ + BzNO_2_^−•^ → ArNO_2_^−•^ + BzNO_2_ for each aromatic nitro compound ArNO_2_, ΔG°_rxn_ = G°_ArNO2−_ + G°_BzNO2_ − G°_ArNO2_ − G_BzNO2−_. Then, we used the Nernst equation to convert to reduction potentials: E° = −ΔG°/nF where n is the number of electrons acquired in the reduction (here = 1) and F is Faraday’s constant. Thus −ΔG°_rxn_/F = E°_rxn_ = E°_ArNO2_ − E°_BzNO2_, and the value of E°’_BzNO2_ = −0.486 V vs. NHE [65,66,95] was used to extract E°_ArNO2_ for each ArNO_2_ at pH 7 (NHE is normal hydrogen electrode). 

Calculations were calibrated against experimental values to compensate for the use of a small basis set and the complete neglect of solvent effects (Supplemental Figure S2). Despite the economy of the computational approach, we obtained a linear correlation of computed to experimental values with R^2^ of 0.96 for Comp = (Exp + 0.379 V)×4.67. We attribute the very large slope to the importance of water’s high dielectric in stabilizing the charge acquired upon reduction, since our calculations do not implement a polarizable continuum dielectric. Similarly, our use of a smaller basis set than those used by highly-accurate computations is likely a reason for the large offset and slope, as larger basis sets are needed to provide a good description of the anionic free radical state formed upon reduction. However, the scatter about the line is small, and after using the equation of the line to calibrate our gas-phase computational values, Calc = Comp/4.67−0.379 V, our ‘Calc’ calibrated calculated E° estimates (E°_c_) display a mean absolute variance of 14 mV from experiment. 

Substrate volumes were calculated in Spartan based on CPK atomic volumes.

### 3.8. Structural Analyses

A total of 36 structures annotated as members of the NR family were gathered from the Protein Data Bank (PDB [96]) based on BLAST-P searches and the set was augmented by sequences identified by BLAST-P in such a way as to broaden representation across subgroups identified by structural motifs. Upon superposition of the structures it was clear that core structure was highly conserved but peripheral structure was not (Supplemental Figure S5). By a stepwise process of identification of structural elements shared by a subset of the structures and removal of that subset followed by re-evaluation and sorting of remaining structures four subgroups emerged, each represented by at least two crystal structures (Figure 6). Several of the structural groups turned out to represent two allied groups in the Akiva/Copp framework. The analysis presented here identifies just one (e.g., NfsB but not MhqN) and adopts the Akiva/Copp nomenclature to promote clarity in the field. Within each subgroup, the peripheral structure corresponded to a similar excursion from the core sequence, which was not identified as such by automated alignments. However, these were folded in the same topology and secondary structure in other members of the subgroup [97]. Based on the structures superimposed in Chimera [98], alignments within and between subgroups were optimized by hand in JALview [99] and Chimera’s multialign viewer. Conserved residues identified were confirmed with those of Akiva/Copp in the Structure Function Linkage Database [48]. Treatment of the peripheral structure as inserts in the sequence resulted alignment of all sequences with far higher homology than if complete sequences are aligned (Figure 5b). Thus, the ‘inserts’ define the distinctions between families. The structure-informed multiple sequence alignment is provided as Supplemental Figure S9.

Buried surface area was obtained using the built-in tool in Chimera and is calculated from the solvent accessible surface area (ASA) per monomer A and B as well as the AB dimer as follows: buried ASA = {ASA(A) + ASA(B) − ASA(AB)}/2 [79] with areas determine as per Lee and Richards [100].

## 4. Conclusions

We have evaluated the roles of nitroaromatic substrate properties and type of NR enzyme as potential determinants of the rate of nitro reduction as well as the capacity to yield amine products. Both factors participate, providing independent degrees of freedom with which systems can be optimized, although no simple single answer emerged. It is evident that members of the NR superfamily offer very wide diversity in their active sites and thus the nature of the contacts between flavin and substrate. This superfamily combines the chemical virtuosity of a flavin with a fold that provides stability from afar via an intertwined structure. The different active site constraints provided by sequence excursions from different portions of the peptide chain produce very different active sites in different NR subgroups. We provide the first correlation between amine production and substrate properties, indicating that the best choices will have large π systems and electron withdrawing substituents. Thus, our work predicts that the most promising NR subgroups for screening and engineering are likely to be those with capacious or flexible active sites such as NfsA, Frm2 and HUB. Our comparisons lay a foundation for chemically and structurally-informed development of modified NRs, exploiting the tolerant architecture and versatile cofactor of this large and diverse superfamily.

## Figures and Tables

**Table 1:** Detection of amine products from compounds modeling substrates reported to undergo reduction to the amine.

#	Compound (ChemSpider ID) ^1^	Product(s)	Analysis ^2^	Detection
**1**	nitrobenzene (7138)	~NHOH	HPLC (40%)	235 & 265 nm
**2**	4-nitrobenzoic acid (5882)	~NHOH	HPLC (30%)	280 nm
**3**	4-nitrobenzenesulfonamide (21360)	~NHOH	HPLC (30%) LC-MS (50%) ~NHOH M−1 = 185	260 nm (−)- mode
**4**	3-trifluoromethyl nitrobenzene (7108)	~NHOH	HPLC (50%)	245 nm
**5**	3-nitrophthalimide (11286)	~NHOH	HPLC (30%) LC-MS (50%) ~NHOH M(+1) = 179 Da	230 nm (+)-mode
**6**	1-nitronaphthalene (6588)	~NHOH	HPLC (40%)	215 nm
**7**	3-nitrofurazone (4566720)	~NHOH, ~NH_2_	HPLC (15%) LC-MS (20%) ~NHOH M(+1) = 185 Da, ~NH_2_ M(+1) = 169 Da	260 & 300 nm (+)-mode
**8**	4-nitro-1,8-naphthalic anhydride (73216)	~NHOH, ~NH_2_	HPLC (50%) LC-MS (50%) ~NHOH M(+1) = 230, ~NH_2_ M(+1) = 214 Da, and [59].	270 & 345 nm (+)-mode
**9**	BTZ043 (24747357)	~NH_2_ ≈ 30% yield	[56]	
**10**	1,3-dinitrobenzene (7172)	3-nitroaniline	[28]	
**11**	CB1954 = 5-(1-aziridinyl)-2,4-dinitrobenzamide (CAS 21919-05-1)	amine products detected	[28]	
**12**	2,4,6-trinitrotoluene (8073)	2- and 4-amino dinitrotoluene	[23,24,25]	

^1^ Information and links on each compound can be retrieved by entering the ChemSpider number as the basis of a search at http://www.chemspider.com/Default.aspx, ^2^ Percentage of mobile phase comprised of acetonitrile (with balance = water). *m*/*z* values are provided for compounds detected using mass spectrometry.

## Supplementary Materials

The following are available online, four tables providing (1,2) lists of compounds studied, (3) surface areas buried in the NR dimers and (4) list of all NR structures and sequences used. Nine figures providing (1) sequence similarity network, (2) calibration and validation of E°_c_ calculations, (3) dependence of amine formation on electron withdrawal (E°_c_), substrate volume or log(P), (4) dependence of rate constants on substituent constants, (5) sequential identification of NR subgroups on the basis of distinguishing structure, (6) numbering of the positions of the flavin right, and that of NADH, (7) comparisons of the shapes and sizes of the different subgroups’ active sites, (8) distribution of amino acid conservation in the structure, and (9) a multiple sequence alignment of the NRs whose structures were compared as well as supporting sequences.
