# Supplementary material for: Informing Efforts to Develop Nitroreductase for Amine Production
Source: Molecules. 2018 Jan 24;23(2):211. doi: 10.3390/molecules23020211 (PMC6017928; doi:10.3390/molecules23020211)
Supplement: Supplementary file 1 [file molecules-23-00211-s001.pdf]

molecules

MDPI

Supplemental Material for"

Informing Efforts to Develop Nitroreductase for Amine Production

Anne-Frances Miller [1,*,#], Jonathan T. Park [2,#], Kyle L. Ferguson [3], Warintra Pitsawong [1] and Andreas S. Bommarius [2,3,*]

[1] Department of Chemistry, University of Kentucky, Lexington, KY, USA; afmill3r2@gmail.com
[2] School of Chemical and Biomolecular Engineering, Georgia Institute of Technology, Atlanta, GA, USA
[3] School of Chemistry and Biochemistry, Georgia Institute of Technology, Atlanta, GA, USA
* Correspondence: afmill3r2@gmail.com or ;andreas.bommarius@chbe.gatech.edu Tel.: +1-859-257-9349

Present addresses:
J. T. Park: Janssen Pharmaceutical Companies of Johnson & Johnson, Malvern, PA
K. L. Ferguson: Dept. of Chemistry, Univ. of Michigan, Ann Arbor, MI.
W. Pitsawong: Dept. Biochemistry, Brandeis University Waltham, MA.

Academic Editor: W. Van Berkel
Received: date; Accepted: date; Published: date

1. Contents

1.1. Tables

S1. Compounds used to calibrate E° calculations, with ChemSpider ID numbers and measured E° values.
S2. Substrates used for kinetic analysis, with ChemSpider ID numbers and Hammett *para* constants (σ).
S3. Surface area (per monomer) buried in dimer interface.
S4. List of the pdb files and amino acid sequences used in the Figures and the sequence alignment

1.2. Figures

S1. Sequence similarity network of the different NR subgroups with ours colored and subgroups containing amine producers identified.
S2. Calibration of calculated E° values vs. experimental values.
S3. Dependence of amine formation on molecular volume and calculated E° or (b) on ** and calculated E°.
S4. Comparison of *St*NfsB vs. *Ms*PnbA via dependence of rates on Hammett *para* coefficient, showing unscaled data and experimental errors.
S5. Visualization of emergence of major subgroups from process of sequential identification of distinguishing elements of structure.
S6. Position numbering for flavin and nicotinamide ring of NADH.
S7. Comparison of the shapes and sizes of binding sites provided by NRs representing each of the four groups.
S8. Maps onto the structure of amino acid conservation within and between the subgroups.
S9. Alignment of the crystallographic sequences showing amine producers.

2. Tables

2.1. Table S1. Compounds used to calibrate E° calculations, and resulting calibrated calculated E° values, with ChemSpider ID numbers and measured E° values.[1]

Calibration Compound	ChemSpider[2]	Uncalibrated Computed E° [3]	Measured E° [1] (mV vs. NHE)	Calculated $E°_c$ [4] (Calibrated)
nitrobenzene	7138	-486	-486	-483
4-chloronitrobenzene	21106020	-231	-450	-428
4-nitroacetophenone	21106581	81	-356	-361
trinitrotoluene	8073	561	-253	-259
3-methyl nitrobenzene	21106146	-556	-475	-498
4-methyl nitrobenzene	13863774	-649	-500	-518
3-chloronitrobenzene	21106013	-188	-405	-419
1,4-dinitrobenzene	7211	551	-257	-261
2,4-dinitrotoluene	8150	41	-397	-370
3-nitroacetophenone	21106145	-185	-437	-419
**Experimental Compound**				
**7** 3-nitrofurazone	4566720		-270 [5]	-349
**Experimental Compound**		**π system** (# atoms)	**Log(P)**	
**1** nitrobenzene	7138	9	0.32	-483
**3** 4-nitrobenzene sulfonamide	21360	12	-1.17	-342
**4** 3-trifluoromethyl nitrobenzene	7108	9	1.24	-392
**5** 3-nitrophthalimide	11286	14	-0.96	-334
**6** 1-nitronaphthalene	6588	13	1.32	-427
**7** 3-nitrofurazone	4566720	14	-2.62	-349
**8** 4-nitro-1,8-naphthalic anhydride	73216	18	0.72	-170
**9** BTZ043	24747357	15	2.01	-259 [6]
**10** 1,3-dinitrobenzene	7172	12	-1.39	-339
**12** 2,4,6-trinitrotoluene	8073	15	-2.61	-259
**13** 3,5-dinitrobenzotrifluoride	109682	12	-0.47	

[1] Measured values were obtained from [1, 2]. Plots are provided as Supplemental Figure S2.

[2] Information and links on each compound can be retrieved by entering the ChemSpider number as the basis of a search at http://www.chemspider.com/Default.aspx

[3] *Ab-initio* computations were used to produce 1-electron *relative* E°s using the functional ωB97X-D and the basis set 6-311+G** as described in methods.

[4] Values calibrated to experiment using the linear dependence in Supplemental Figure S2(**a**): Calibrated = (Computed + 379)*4.667.

[5] The measured E° for 3-nitrofurazone at 7.45, which corresponds to ~NO_2 + $2e^-$ + $2H^+$ → ~NO_2 + H_2O [3].

[6] Computations of E° retained the full aromatic system and attached amine but replaced the aliphatic extension with two Hs.

2.2. Table S2. Substrates used for kinetic analysis, with ChemSpider ID numbers and Hammett *para* constants (σ). Plots are provided as Supplemental Figure S4.

			***St*NfsB**		***Ms*PnbA**	
**Compound**	**Chem-Spider**[1a]	**Hammett σ para** 2	k_{cat} (s^{-1})	k_{cat}/K_M ($s^{-1}.mM^{-1}$)	k_{cat} (s^{-1})	k_{cat}/K_m ($s^{-1}.mM^{-1}$)
nitrobenzene	7138	0	-	1.7 ± 0.1	3.4 ± 0.4	1.2 ± 0.3
4-nitrobenzoate	3620348	0	5.8 ± 0.3	5.1 ± 0.6	-	-
4-chloronitrobenzene	21106020	0.227	-	5.2 ± 0.2	4.5 ± 0.5	11 ± 4
methyl-4-nitrobenzoate	11586	0.45	-	84.5 ± 6.8	9.0 ± 0.3	75 ± 7
4-nitroacetophenone	21106581	0.5	166 ± 25	167 ± 54	-	-
4-nitrobenzenesulfonamide	21360	0.6	182 ± 11	108 ± 20	17.2 ± 0.9	91 ± 13
4-nitrobenzonitrile	11593	0.66	290 ± 13	238 ± 22	16.0 ± 1.0	111 ± 22
methyl 4-nitrobenzenesulfonate	21173	0.9	275 ± 22	373 ± 90	19.1 ± 0.8	165 ± 18

[1] Information and links on each compound can be retrieved by entering the ChemSpider number as the basis of a search at http://www.chemspider.com/Default.aspx

[2] [4]

2.3. Table S3. Surface area (per monomer) buried in dimer interface.

**Subgroup**	**Instance** (PDB ID)	**Buried Area** ($Å^2$)[1]	**Statistics** ($Å^2$)
NfsB	5J8D	4907, 5225	Avg = **4600** ± 400
	3HZN	4137	
	1YKI	4681, 4849	
	1VFR	4135	
	3OF4	4321	
	2HAY	4708	
NfsA	1F5V	5751	Avg = **5500** ± 300
	2BKJ	4892	
	3N2S	5599, 5593	
	3EOF	5787	
PnbA	2WZW	5014	Avg = **5080** ± 70
	3GR3	5146	
Frm2	2IFA	4050, 3957, 3806	Avg = **3900** ± 100
HUB	3E39	4168	Avg = **4200** ± 100
	3E10	4372	
	4DN2	4172	

[1] Average of areas buried of each of two participating monomers, multiple values correspond to area buried in AB dimer, CD dimer (if present), EF dimer (if present). Calculated in Chimera [5].

2.4. Table S4. List of the pdb files and amino acid sequences used in the Figures and the sequence alignment in Figure S9.

**Accession code**[1] (ID in MSA)[a]	**Species source and annotated name**	Subgroup[2], Amine production and ref.
Cons_NfsB	*NONE*<br>consensus sequence for NfsB from SFLD[3]	
5J8D<br>Ent_5J8D	*Enterobacter cloacae* NR UniProtKB AC: Q01234	NfsB
1YKI<br>Ecoli_1YKI	*Escherichia coli* NfnB UniProtKB AC: P38489	NfsB
1VFR<br>Vfisch_1VFR	*Vibrio fischeri* major NAD(P):FMN oxidoreductase UniProtKB AC: P46072	NfsB
3QDL<br>Helicobac_3QDL	*Helicobacter pylori* RdxA UniProtKB AC: O25608	NfsB
3OF4<br>Idiomar_3OF4	*Idiomarina loihiensis* L2TR Nitroreductase/dihydropteridine reductase UniProtKB AC: Q5R179	NfsB
3HZN<br>Styph_3HZN	**Salmonella typhinurium *dihydropteridine reductase*** ***UniProtKB AC: P15888***	***NfsB AMINE (this work)***
2HAY<br>Spyo_2HAY	*Streptococcus pyogenes* M1 GAS Putative NAD(P)H-Flavin Oxidoreductase UniProtKB AC: Q9A120	NfsB
3BEM<br>Bsubt_3BEM	*Bacillus subtilis* putative nitroreductase YdfN (2632848) UniProtKB AC: P96692	MhqN
4QLX<br>Lplant_4QLX	*Lactobacillus plantarum* enone reductase UniProtKB AC: U6C5W9	MhqN
3GBH<br>Sepider_3GBH	*Staphylococcus epidermidis* ATCC 12228 putative NAD(P)H:FMN oxidoreductase (SE1966) UniProtKB AC: A0A0H2VHN8	MhqN
3GE6<br>Esibir_3GE6	*Exiguobacterium sibiricum* 255-15 putative nitroreductase EXIG_2970 UniProtKB AC: B1YG32	MhqN
AAP09971<br>AMINE_Bcer3024	*Bacillus cereus* ATCC 14579<br>NAD(P)H nitroreductase called BC_3024 and YdgI_Bc	MhqN, AMINE inferred [6]
3GAG<br>Smutants_3GAG	*Streptococcus mutans* nitroreductase-like protein (smu.346) UniProtKB AC: Q8DVW4	MhqN
2B67<br>Spneum_2B67	*Streptococcus pneumoniae* TIGR4 nitroreductase family protein UniProtKB AC: A0A0H2UP38	MhqN
Cons_NfsA	*NONE*	

	consensus sequence for NfsA from SFLD	
1F5V<br>Ecoli_1F5V	*Escherichia coli* NfsA<br>UniProtKB AC: P17117	NfsA
2BKJ<br>Vharv_2BKJ	*Vibrio harveyi* Flavin reductase<br>UniProtKB AC: Q56691	NfsA
3N2S<br>Bsubt_3N2S	*Bacillus subtilis*<br>NfrA1 nitroreductase<br>UniProtKB AC: P39605	NfsA AMINE [7]
3EOF<br>Bfrag_3EOF	Bacteroides fragilis<br>Oxidoreductase (YP_213212.1)<br>UniProtKB AC: Q5L9C9	NfsA
KXV33494<br>AMINE_Gox0834	*Gluconobacter oxydans* Nitroreductase Gox0834	NfsA AMINE [8]
AAP08598<br>AMINE_Bcer1619	*Bacillus cereus* ATCC 14579<br>Oxygen-insensitive NADPH nitroreductase called BC_1619 or YfkO_Bc	NfsA, AMINE inferred [6]
010964037<br>AMINE_CaceNit<br>A	*Clostridium acetobutylicum* 824<br>NitA WP_010964037 NADPH-dependent oxidoreductase	NfsA AMINE [9]
Cons_PnbA	*NONE*<br>consensus sequence for PnbA from SFLD	
2WZW<br>AMINE_Msmg_2<br>WZW	*Mycobacterium smegmatis*<br>Nitroreductase NfnB<br>UniProtKB AC: A0R6D0	PnbA, AMINE [10]
3GR3<br>Bhen_3GR3	*Bartonella henselae* str. houston-1<br>PnbA<br>UniProtKB AC: A0A0H3M323	PnbA
WP_031019703.1<br>Strep	*Streptomyces* sp. NRRL WC-3795<br>Nitroreductase	PnbA
WP_055507769.1<br>Saur	*Streptomyces aurantiacus*<br>Oxidoreductase	PnbA
WP_080719519.1<br>Coryn	*Corynebacterium jeikeium*<br>Nitroreductase	PnbA
WP_014983339.1<br>Nocar	*Nocardia brasiliensis*<br>Oxidoreductase	PnbA
WP_043519713.1<br>Kpneu	*Klebsiella pneumoniae*<br>Nitroreductase	PnbA
WP_057084594.1<br>Dicke	*Dickeya solani*<br>Nitroreductase	PnbA
WP_076001261.1<br>Halio	*Halioglobus pacificus*<br>Nitroreductase	PnbA

KPQ07647.1<br>Rhodo	*Rhodobacteraceae bacterium* HLUCCA12<br>Nitroreductase	PnbA
ONF96818.1<br>Sphin	*Sphingomonas* sp. G39<br>Coenzyme F420:L-glutamate ligase	PnbA
WP_014992242.1<br>Actino	*Actinobacillus suis*<br>Nitroreductase	PnbA
Cons_Frm2	*NONE*<br>consensus sequence for Frm2 from SFLD	
2IFA<br>Smut_2IFA	*Streptococcus mutans*<br>Putative nitroreductase<br>UniProtKB AC: Q8DW21	Frm2
4URP<br>Scere_4URP	*Saccharomyces cerevisiae*<br>Nitroreductase<br>UniProtKB AC: P37261	Frm2
2WQF<br>Llact_2WQF	*Lactococcus lactis*<br>Nitroreductase CinD<br>UniProtKB AC: Q9CED0	Frm2
1YWQ<br>Bcer_1YWQ	*Bacillus cereus* ATCC 14579<br>Nitroreductase family member<br>UniProtKB AC: Q81EW9	Frm2
Cons_HUB	*NONE*<br>consensus sequence for HUB from SFLD	
3E39<br>Desulf_3E39	*Desulfovibrio desulfuricans* putative nitroreductase dde_0787<br>UniProtKB AC: Q314Q8	HUB
3E10<br>Cace_3E10	*Clostridium acetobutylicum* putative NADH oxidase NP_348178.1<br>UniProtKB AC: Q97IT9	HUB
WP_010966820<br>AMINE_CaceNit<br>B	*Clostridium acetobutylicum*<br>NitB nitroreductase	HUB AMINE [9]
3KWK<br>Bthet_3KWK	*Bacteroides thetaiotaomicron* putative nitroreductase NP_809094.1<br>UniProtKB AC: Q8ABC9	HUB
3GE5<br>Pging_3GE5	*Porphyromonas gingivalis* w83<br>Putative NAD(P)H:FMN oxidoreductase<br>UniProtKB AC: Q7MX99	HUB
3G14<br>Cnov_3G14	*Clostridium novyi* NT Nitroreductase family protein YP_877874.1<br>UniProtKB AC: A0PZS2	HUB
4DN2	*Geobacter metallireducens* GS-15 putative	HUB

Gmetal_4DN2	nitroreductase<br>UniProtKB AC: Q39RS1	
3GFA<br>Cdiff_3GFA	*Clostridium difficile* 630 putative nitroreductase YP_001089721.1<br>UniProtKB AC: Q17ZU8	HUB
Tther_1NOX	*Thermus thermophilus* NADH oxidase<br>UniProtKB AC: Q60049	HUB
3EO8<br>Cdiff_3EO8	*Clostridium difficile* 630<br>BluB-like flavoprotein YP_001089088<br>UniProtKB AC: Q182R2	HUB
Mtub_4XOM<br>Mtubr_4XOM	*Mycobacterium tuberculosis* C-terminal domain of CoenzymeF420:L-glutamate ligase<br>UniProtKB AC: P9WP79	FbiB
4EO3<br>Tmar_4EO3	*Thermotoga maritima*<br>Peroxireductase nitroreductase fusion enzyme. UniProtKB AC: Q9WYL7	TdsD
2FRE<br>Agro_2FRE	*Agrobacterium tumefaciens* (fabrum)<br>Oxidoreductase<br>UniProtKB AC: A9CKT4	TdsD
AFW02496<br>Gox	*Gluconobacter oxydans* H24<br>Nitroreductase	TdsD
3GB5<br>Mmus_3GB5	*Mus musculus* iodotyrosine deiodinase IYD<br>UniProtKB AC: Q9DCX8	Iyd
4TTC<br>Hsap_4TTC	*Homo sapiens* iodotyrosine deiodinase IYD<br>UniProtKB AC: Q6PHW0	Iyd
3EK3<br>Bfrag_3EK3	*Bacteroides fragilis* NCTC 9343 Nitroreductase YP_211706.1<br>UniProtKB AC: Q5LDN3	Unknown 2
3PXV<br>Dhafn_3PXV	*Desulfitobacterium hafniense* DCB-2 Nitroreductase<br>UniProtKB AC: B8FRE0	Unknown 2
2ISJ<br>Smel_2ISJ	*Sinorhizobium meliloti* BluB<br>Flavin conversion to 5,6-dimethylbenzimidazole.<br>UniProtKB AC: Q92PC8	BluB
3K6H<br>Agro_3K6H	*Agrobacterium tumefaciens* Str. C58 (fabrum)<br>Nitroreductase UniProtKB AC: A9CIP6	Unknown 1

[1] PDB IDs are provided if applicable. For sequences not associated with a structure the NCBI accession number is provided and in parenthesis the abbreviation used in the Multiple Sequence Alignment. Accession codes can be associated with further information by using them as the basis for a search at https://www.ncbi.nlm.nih.gov/home/proteins/ courtesy of the National Center for Biotechnology Information, U.S. National Library of Medicine 8600 Rockville Pike, Bethesda MD, 20894 USA

[2] Subgroups are those of Akiva/Copp [11]. Enzymes for which we found literature reports of amine production are so indicated with the reference.

[3] SFLD is the Structure-Function Linkage Database [12].

3. Figures

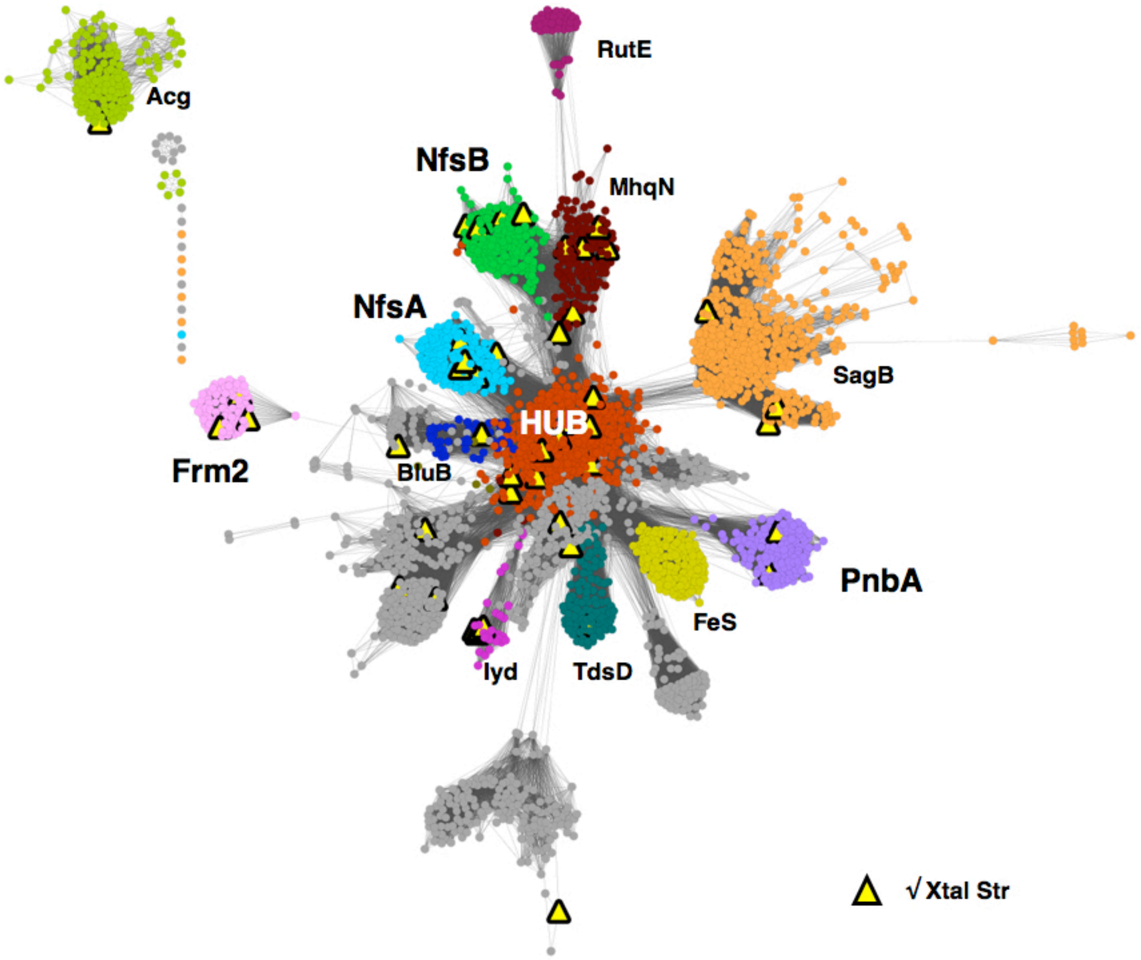

3.1. Figure S1. Sequence similarity network of the different subgroups of the nitroreductase superfamily coloured similarly to Akiva, Copp et al [11] but with unknown subgroups in grey. Small differences in the distribution of the subgroups have resulted from the use of a different software but the topology of the network is unchanged (compare with Figure 2 of [11]). Each small coloured or grey dot is a node of amino acid sequences sharing 50% or higher identity. Any notes sharing homology scoring higher than e^{-16} with another node is connected to it with a line. Those nodes that include a solved crystal structure are depicted as yellow triangles with heavy borders. In each subgroup with ours colored and subgroups containing amine producers identified. Otherwise each node is coloured according to the subgroup to which it is assigned. The colours replicate those of Akiva/Copp except where needed to permit use of the same colours in structural figures. Thus rust replaces the red of Akiva/Copp in order to allow CPK to be used (red = oxygen); bright blue replaces light teal for NfsA, the pale mauve (PnbA) and rose (Frm2) of Akiva/Copp have also been rendered in more saturated colours, to improve structural figures. For the origins of subgroup names, see Akiva, Copp et al. [11].

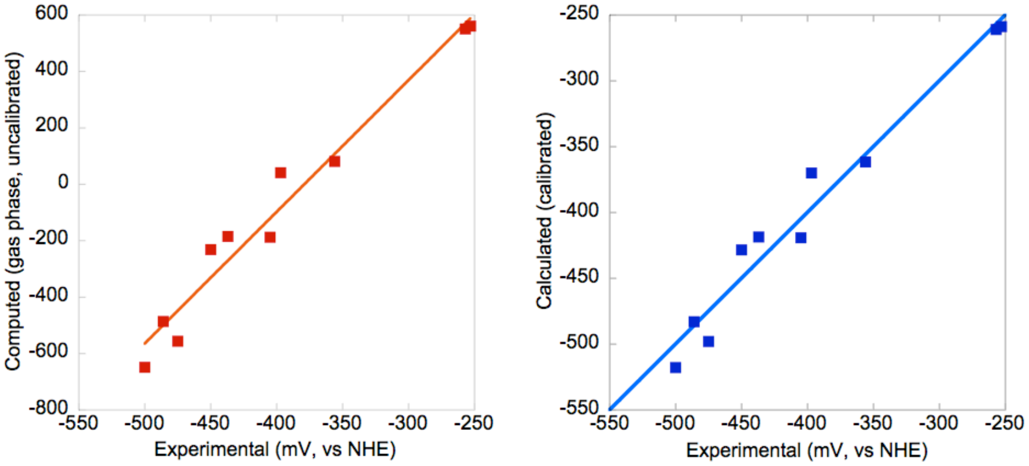

(a) (b)

3.2. Figure S2. (**a**) Calibration of calculated E° values *vs*. experimental values and (**b**) resulting correspondence between calibrated calculations and experiment yielding MAD = 14 mV. *Ab-initio* computations were used to produce 1-electron *relative* E°s using the functional ωB97X-D and the basis set 6-311+G** as described in methods. These computed values of E° show a linear trend vs. experimental values with R^2 = 0.96 and Comp = (Exp + 379)*4.67 where 'Comp' is the value from *ab initio* gas-phase calculation in mV. The 379 mV systematic offset and the multiplier of 4.67 both likely arise from our use of gas-phase calculations and a modest level of theory whereas the measured values derive from water (for another example see [1, 13]). Thus we emphasize that the *ab-initio* calculations at our level of theory in gas phase do not themselves yield values that replicate experiment in water. They do however produce a tight and linear correlation with all 10 experimental values that were most pertinent to the class of molecules and reaction under study, and therefore permit estimations of anticipated E°s where they are not available from experiment. Thus we treated the straight-line correlation as a calibration curve, and applied the calibration to obtain calibrated values Cal = (Comp/4.67)-379 to produce calculated 1-e E°s that are calibrated against experiment. This compromise has allowed us to apply the method to larger molecules than would have been accessible without access to specialized resources. The success of the calculated results can be assessed by comparing the plot vs. experimental data (dark blue squares) with an Y=X line. The mean absolute deviation (MAD) of the calculated values from the line is 14 mV.

Compounds used are tabulated in Supplemental Table S2 with measured values obtained from [1, 2].

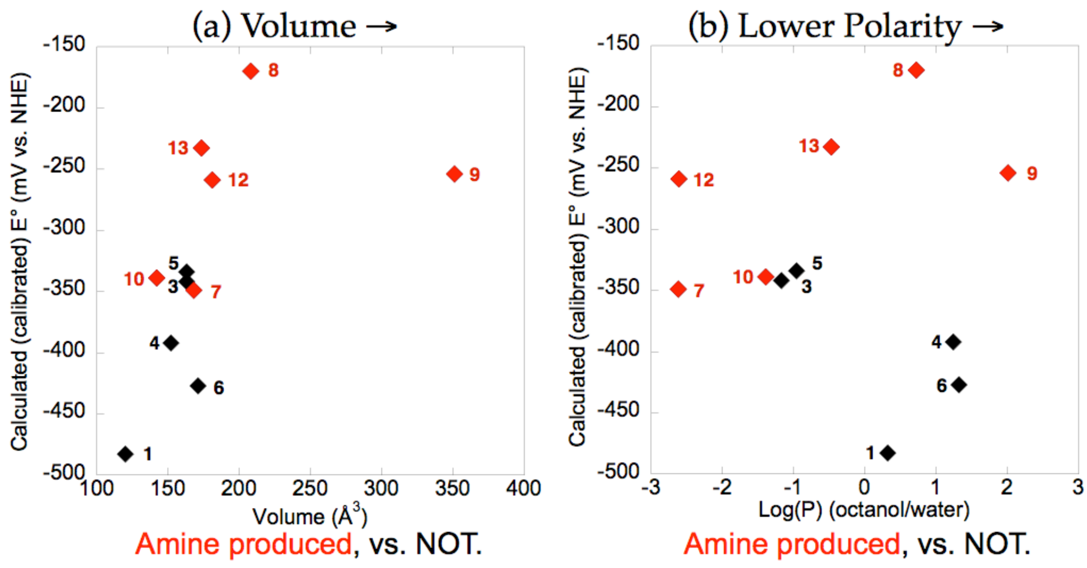

3.3. Figure S3. (**a**) Dependence of amine formation on molecular volume and calculated E° or (**b**) on log(P) and calculated E°. Volume and log(P) were calculated in Spartan based on CPK atomic volumes and an estimated octanol/water partition coefficient calculated by the method of Ghose and Crippen. While larger molecules (right hand side of panel (a)) seem slightly more prone to conversion to amines, molecular polarity does not appear to correlate with propensity to form amines (**b**). In both panels the vertical axis is calculated E°, which correlates with formation of amine product.

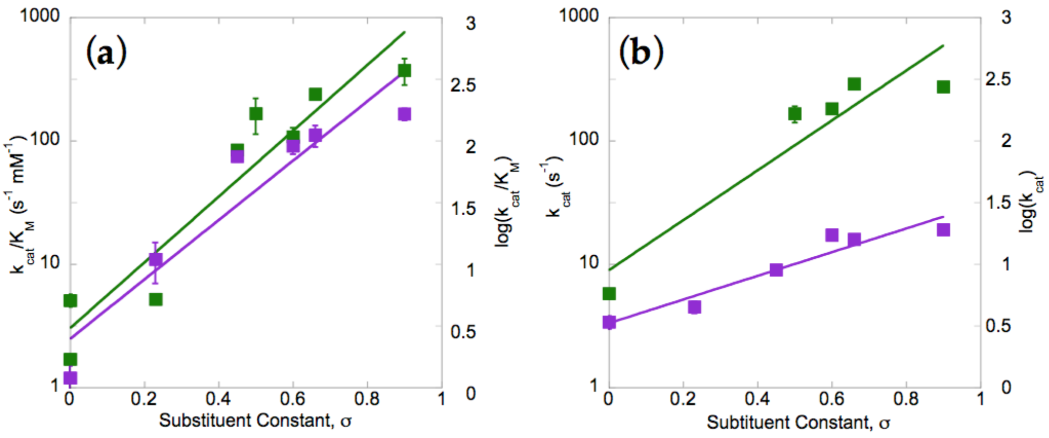

3.4. <u>Figure S4</u>. Plots of enzymatic reaction rates showing the errors evaluated via triplicate measurements for *St*NfsB (green) and *Ms*PnbA (purple). Data are plotted with respect to sensitivity to Hammett para constant σ [4]. (**a**) shows dependence of the second-order rate constant and (**b**) shows that of the first-order rate constant. (**b**) here is the same as panel (**b**) of Figure 3, but is reproduced here to permit direct comparison with panel (**a**). Note that error bars are shown but rarely exceed the size of the symbol. Note also that these data are not normalized to the rate constant of the unsubstituted parent compound but presented as measured to permit direct comparison between the rates produced by the two different enzymes. Log(k_{cat}/K_M) data are fit with lines, with for *St*NfsB a slope of 2.7± 0.4 and an intercept of 0.5 ± 0.2 (R^2=0.89), and for *Ms*PnbA a slope of 2.4 ± 0.4 and intercept of 0.4 ± 0.2 (R^2=0.88). Log(k_{cat}) data are fit with lines, with for *St*NfsB a slope of 2.0 ± 0.4 and an intercept of 0.9 ± 0.3 (R^2=0.88), and for *Ms*PnbA a slope of 1.0 ± 0.1 and intercept of 0.5 ± 0.08 (R^2=0.92). Values are tabulated in Supplementary Table S1.

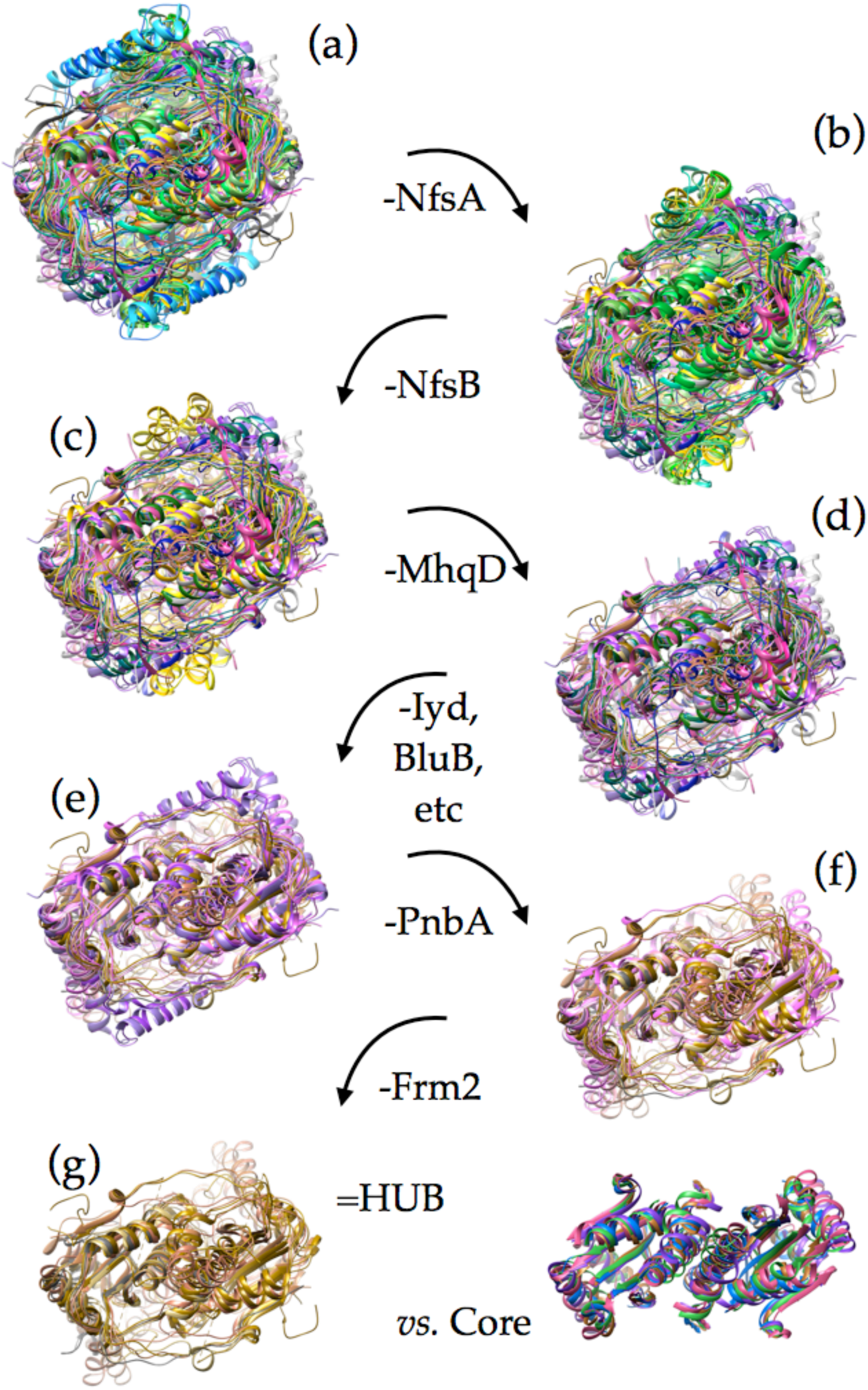

3.5. Figure S5. View of steps in which successive subgroups can be identified and removed from the remaining large collection of NR-related enzyme structures. Panel A is the overlay of 36 structures shown in the manuscript, in B the NfsA structures have been removed, in C the NfsB structures have additionally been removed, D further lacks the structures of MhqD subgroup

members, and so forth. Finally only the structures of HUB subgroup members remain. PDB accession numbers of all the structures are in Supplementary Table S4.

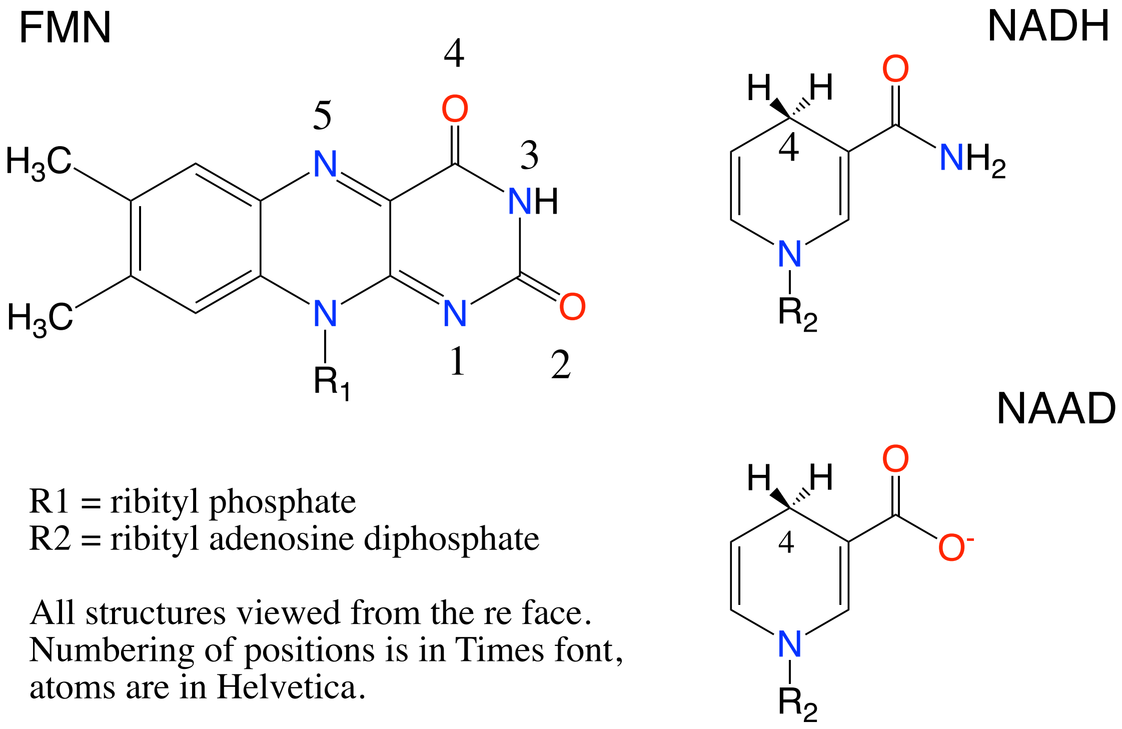

3.6. Figure S6. Position numbering for flavin, nicotinamide ring of NADH and analogous nicotinic acid ring of the nicotinic acid adenine dinucleotide used as a model for NADH in crystallography of *Ent*NfsB.

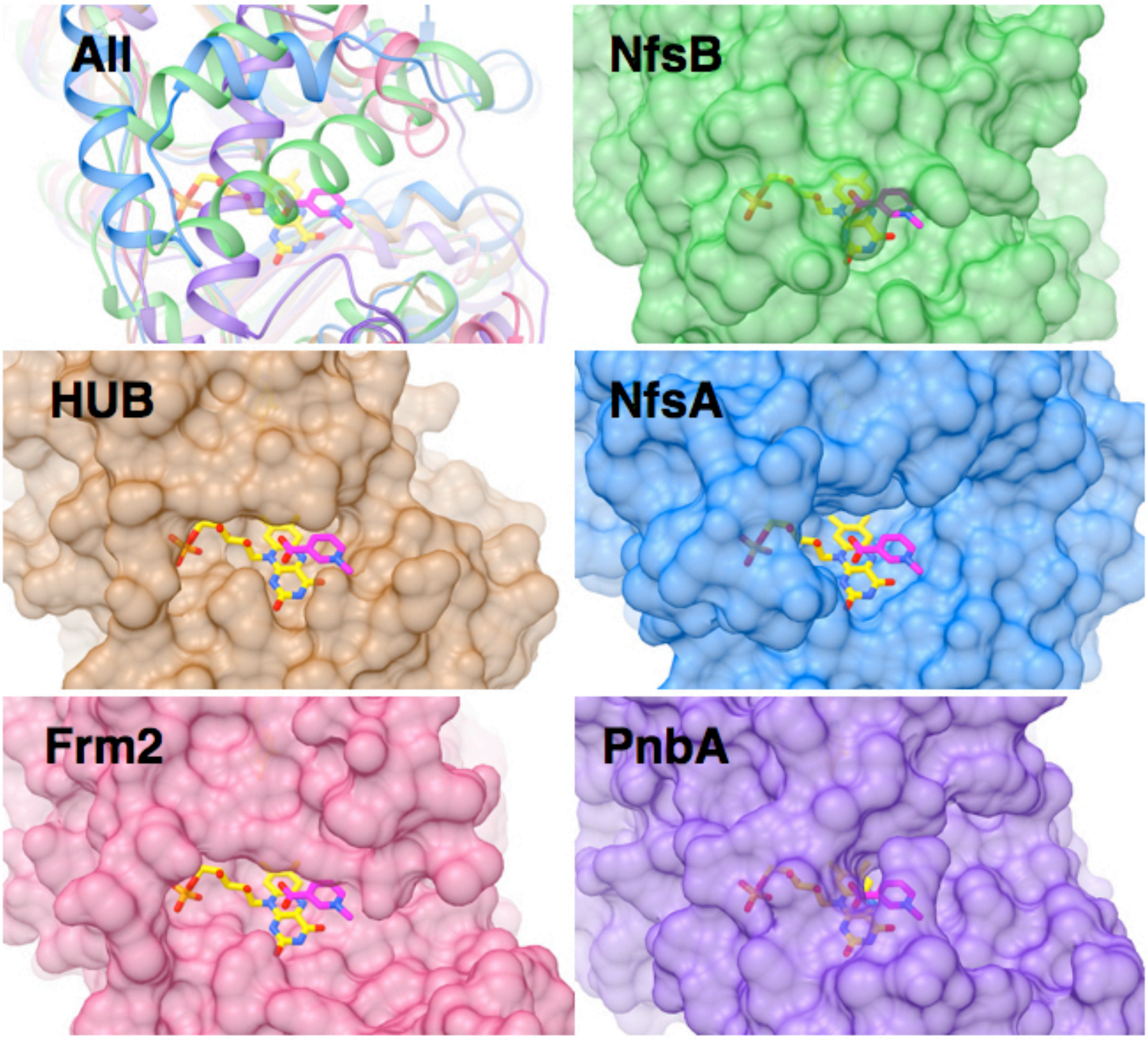

3.7. Figure S7. Comparison of the shapes and sizes of binding sites provided by NRs representing each of the four groups. Overlay of the ribbon structures of *Ms*PnbA (purple, 2WZV), *St*NfsB (green, 5J8D), *E. coli* NfsA (blue, 1F5V), *Smut* Frm2 (pink, 2IFA), *Desulf* HUB (brown, 3E39). In each case one of the fine structures' surface is shown at 50% transparency, and the ribbons of all structures are shown for reference in the top left panel. The FMN and the nicotinic acid ring of bound NAAD of 5J8D are shown in all cases to provide markers for the active site. NAAD is nicotinic acid adenine dinucleotide, and analog of NADH (Supplemental Figure S6).

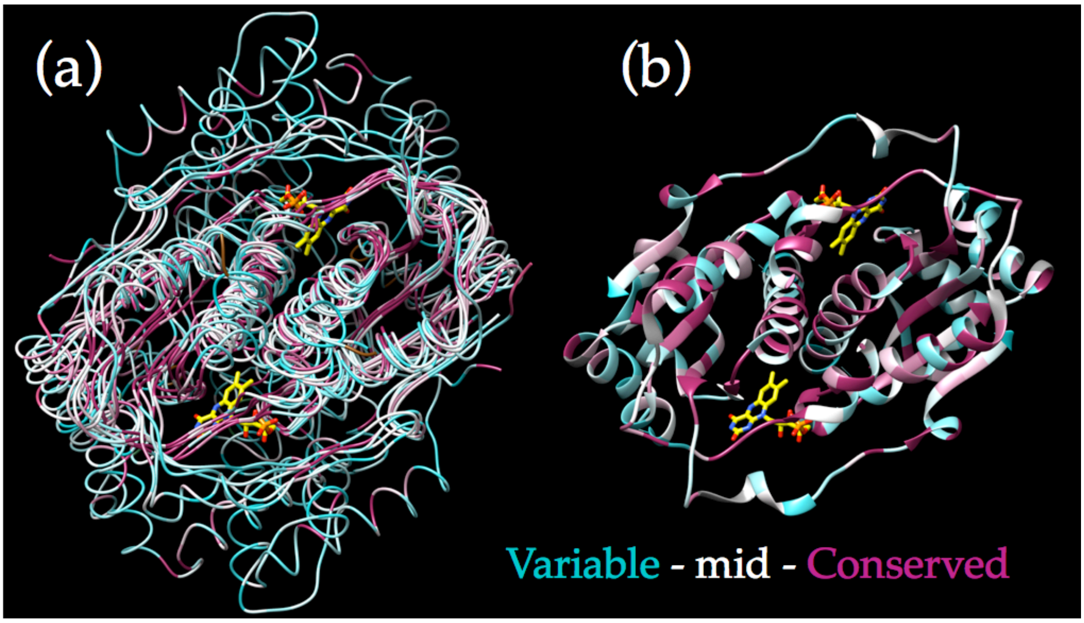

3.8. Figure S8. Comparison of (**a**) distribution of conservation within each of the subgroups - each depicted as a cord, with (**b**) conservation between subgroups depicted on core sequence only (ribbons). Subgroups included are NsfA, NsfB, PnbA, Frm2, HUB. Conservation at each residue was provided from multiple sequence alignment of each subgroup provided by the Structure Function Linkage Database [12].

3.9. Figure S9. Following 5 pages:

Alignment of the crystallographic and supporting other amino acid sequences showing amine producers. The representative sequence from each subgroup is labeled on a coloured background with the theme that has been used throughout: NfsB (Ent_5J8D, green), NfsA (Ecoli_1F5V, blue), Frm2 (Smut_2IFA, pink), PnbA (AMINE_Msmg_2WZW, purple) and HUB (Desulf_3E39, rust). In the alignment, secondary structure is enclosed in yellow boxes (helices) and green (beta strands).

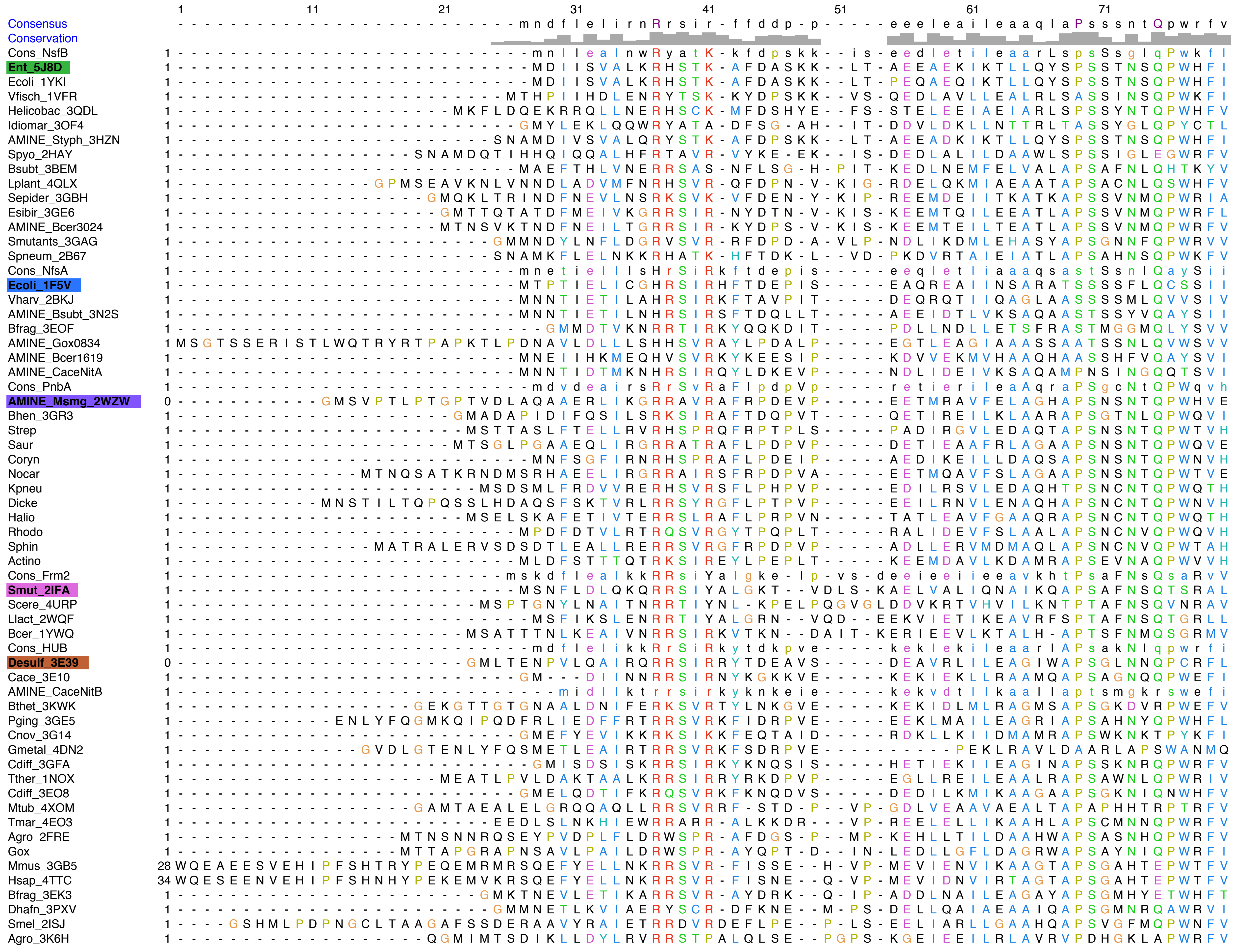
1
11
21
31
41
51
61
71
Consensus
Conservation
Cons_NsfB
Ent_5J8D
Ecoli_1YKI
Vfisch_1VFR
Helicobac_3QDL
Idiomar_3OF4
AMINE_Styph_3HZN
Spyo_2HAY
Bsubt_3BEM
Lplant_4QLX
Sepider_3GBH
Esibir_3GE6
AMINE_Bcer3024
Smutants_3GAG
Spneum_2B67
Cons_NfsA
Ecoli_1F5V
Vharv_2BKJ
AMINE_Bsubt_3N2S
Bfrag_3EOF
AMINE_Gox0834
AMINE_Bcer1619
AMINE_CaceNitA
Cons_PnbA
AMINE_Msmg_2WZW
Bhen_3GR3
Strep
Saur
Coryn
Nocar
Kpneu
Dicke
Halio
Rhodo
Sphin
Actino
Cons_Frm2
Smut_2IFA
Scere_4URP
Llact_2WQF
Bcer_1YWQ
Cons_HUB
Desulf_3E39
Cace_3E10
AMINE_CaceNitB
Bthet_3KWK
Pging_3GE5
Cnov_3G14
Gmetal_4DN2
Cdiff_3GFA
Tther_1NOX
Cdiff_3EO8
Mtub_4XOM
Tmar_4EO3
Agro_2FRE
Gox
Mmus_3GB5
Hsap_4TTC
Bfrag_3EK3
Dhafn_3PXV
Smel_2ISJ
Agro_3K6H

```
                        81        91        101       111       121       131       141       151
Consensus                  vvsg----k-rlalal-aa--------------------------------------------------------f-ga-
Conservation
Cons_NsfB          50  vven-pelkek-lk---paaw--------------------------------------------------nqpqitdaS
Ent_5J8D           50  VAST-EEGKARVAKSA-AGTY------------------------------------------------VFNERKMLDAS
Ecoli_1YKI         50  VAST-EEGKARVAKSA-AGNY------------------------------------------------VFNERKMLDAS
Vfisch_1VFR        52  VIES-DAAKQRM-HDS-FANM-----------------------------------------------HQFNQPHIKACS
Helicobac_3QDL     56  MVTD-KDLKKQIA-A--HSYF--------------------------------------------------NEEMIKSAS
Idiomar_3OF4       50  VIRN-KGLREQLVN---HSF---------------------------------------------------GQQKVADSS
AMINE_Styph_3HZN   53  VAST-EEGKARVAKSA-AGNY------------------------------------------------TFNERKMLDAS
Spyo_2HAY          58  VLDN-KPIKEEI-KP--FAWG--------------------------------------------------AQYQLETAS
Bsubt_3BEM         51  TVLDQDV-KEKLKQ--AANG----------------------------------------------------QYKVVSSS
Lplant_4QLX        62  VVDTPEA-KAKFKQAVMKFN----------------------------------------------------YPQVDSAS
Sepider_3GBH       58  VVQSDEM-KEKVKE-SFGFN----------------------------------------------------SRQLTTSS
Esibir_3GE6        57  VIDSEEG-KATLAP-LAKFN----------------------------------------------------QVQVETSS
AMINE_Bcer3024     57  VIESDEA-KATLAP-LAKFN----------------------------------------------------QSQVETSS
Smutants_3GAG      53  VVKNKNK-QEDLKK-LAALQ-----------------------------------------------------PQVATAS
Spneum_2B67        52  VVREK---NAELAKLAYGSN------------------------------------------------------FEQVSS
Cons_NfsA          50  rvtd---pekkarLaelag--------------------------------------------------nQpyvaqap--
Ecoli_1F5V         50  RITD---KALREELVTLTG--------------------------------------------------GQKHVAQAA--
Vharv_2BKJ         50  RVTD---SEKRNELAQFAG--------------------------------------------------NQAYVESAA--
AMINE_Bsubt_3N2S   50  GVSD---PEKKRELSVLAG--------------------------------------------------NQPYVEKNG--
Bfrag_3EOF         48  VTRD---AEKKEILSPAH-F-------------------------------------------------NQPMVKEAP--
AMINE_Gox0834      76  AVEN---PETRARLAELCG--------------------------------------------------NQKHIEVAP--
AMINE_Bcer1619     50  YVTD---QDLKIKLAELSG--------------------------------------------------N-RHVKDCA--
AMINE_CaceNitA     50  VVRD---KKKREKLAELVG--------------------------------------------------NQEYVAKAP--
Cons_PnbA          49  vvtG--ealdrlraalleaaaagepspdfpypr-ey-rgpyreRrrevgwqlygalGlargDkeararqalrnfrfFgAP
AMINE_Msmg_2WZW    64  VVSG--AARDRLAEALVTAHAEERVTVDFPYREGLFQG-VLQERRADFGSRLYAALGIARDQTDLLQGYNTESLRFYGAP
Bhen_3GR3          55  VLTG--KILQKVGQELSQLVLSGIKGEREYHYYPRQWREPYLSRRRKVGLDLYKSLGIQKGDQEKMLHQKAKNFLFYGAP
Strep              54  IVSG--AA--RDALSKELLKAYEDGRDSADFTGDYGDG-VYLERSYQHGARVYGARGIARSDMEGRNEVIRENLRFYDAP
Saur               55  VASG--AARDNLAEALVAAHRAGRRTVDFPFSEDTFTP-VHQHRRAAMGAALYGALGIDRDDHELRAAYDVQSLSFYGAP
Coryn              49  VIGG--QKL-KDLSAALIEEFDTNGLNPDFTIDYGQGV-HPQ-RSMELAAKMYGLLGIAREDKEARTEFVRENLRFFGAP
Nocar              62  VVSG--PARDRLSAALLTAHTQKYLSVDFPYRDDLYTD-LHRRRREDFGETVYRALGIARDDHPAREAHSTESLRFYGAP
Kpneu              53  IVSG--SKKDVLSKAILAADDAGQLTPDFSFGINDFPG-VYRERYKEQGAAYYQAIGVKRDDKDLRREASRRNLEFFGAP
Dicke              65  IVSG--KKLTELAKILHEKNNAGELTPDFTFDMDHFYG-AYGERKNAQGKAYYEAMNIAREDKEGRHRAVAYNYSFFNAP
Halio              54  VASG--DAVETLRKEMPSRFMQGEMSLDFPYDGVY-EG-VYKQRQHGSAQALYDAAGIAREDKARRHEQFMENFTFFGAP
Rhodo              50  VVSR--AVLRALGHAMTEAARSGQPPAPDVPMISRYEG-PFRHRQIDAAKALYGAMGIARNDHDGRAAAFLRNLDAFGAP
Sphin              61  VVSG--EAAERMRTVLHDAAKSGTKMTPDFPLTGAYPG-HYRGRQIDAAKSLFAATNVARDDVPARTESFLRNFRFFDAP
Actino             50  IVSG--EAL-KRLSDKLVDGFRTGNLAPDFIFDQHALTGVYEDRMRDSYKRLYDAFNILREDKAGRKSFAEENARFYGAP
Cons_Frm2          53  vLfg--eehdklWd-itketLr-kivpaeaf-----------------------------------aaTeeKidsFkag-
Smut_2IFA          52  VLFG--QDSQDFWNKIAYSELE-KVTPAEAF-----------------------------------AGTKAKLESFAAG-
Scere_4URP         57  IIVG--DTHKRIWDAVA-SAMP---T----------------------------------------AEAKKRPESCRDEA
Llact_2WQF         50  ILTG--DAQDKLWDEIVAPELK--AAMEAQG-----------------------------VPESAWDNTRAKLDGFK-AA
Bcer_1YWQ          55  VLMD--GEHEKFWD-IVKETLRARV-PAENF-----------------------------------EATVERLKGFHAG-
Cons_HUB           49  vvt---d--k-ell-e--kl-----a-----------------kp----------------------------yg-amfk
Desulf_3E39        53  VIRAD-D-PRCDILAA--HTR----------------------------------------------------YG-HIVR
Cace_3E10          47  VLE-D-R-ENIDKLSN--FSK----------------------------------------------------YA-NSLK
AMINE_CaceNitB     47  avtn-knliselsta---------------------------------------------------rkmgsq-f----lk
Bthet_3KWK         58  VVS-D-R-AKLDSMAA-ALP-----------------------------------------------------YA-KMLT
Pging_3GE5         64  VVR-E-E-EGRKRLAP--CS-----------------------------------------------------QQPW-FP
Cnov_3G14          50  VVE-S-D-KLKLDIAN-AIENK----------------------TS---------------------------AASEAVL
Gmetal_4DN2        57  CWRFV-V--VE-DQAT-KVQISELSYVEAYFGP-----------------------------------------------
Cdiff_3GFA         49  VIT---E-KEKESMLK-AMSKGIQNEINDNGLLPGSR-Q-HIAGAN---------------------------YTVEIMK
Tther_1NOX         56  VVR-D-P-ATKRALRE-AAFG----------------------------------------------------QA-HV-E
Cdiff_3EO8         50  VIK-R-R-DLMEKIAD-VITKKQQEILVEMDKVSVDKANRFRKFVK---------------------------NFTLFYL
Mtub_4XOM          58  WL-QTPAI-RARLLDRMKDKWRSDLTSDGLPADAIERR-VARGQILY------------------------------DAP
Tmar_4EO3          52  VVDEEEL--------LKKIHE---------------ALPG-----------------------------------GNYWM
Agro_2FRE          59  YAHKDSE-------DWPLF-----------------VELLME--------------------------------GNQKWA
Gox                59  YARRNTPD-------WDRF-----------------LSWLIPF--------------------------------NRSWA
Mmus_3GB5         103  VVKDPDM--------KHKIREIIEEEEEINYMKRMGKRWVTDLKKLRTNWIKEYLD------------------------
Hsap_4TTC         109  VVKDPDV--------KHKIRKIIEEEEEINYMKRMGHRWVTDLKKLRTNWIKEYLD------------------------
Bfrag_3EK3         53  AVCNTVKLEEL----NERIKGAFAKSDDKHLRERGHSETYCCYYHA----------------------------------
Dhafn_3PXV         52  VVKNKEL--------MQEMEA-----------EGLAYLAGMEDQSSYNRIMERGGR---------------------LFY
Smel_2ISJ          73  LVRQDET--------REKV-----------------WQ---AF----------------------------------QRA
Agro_3K6H          59  VYRGEE---------RVRL-----------------------SEAALRIALEKNPDLDLQQQEAERT----------RFT
```

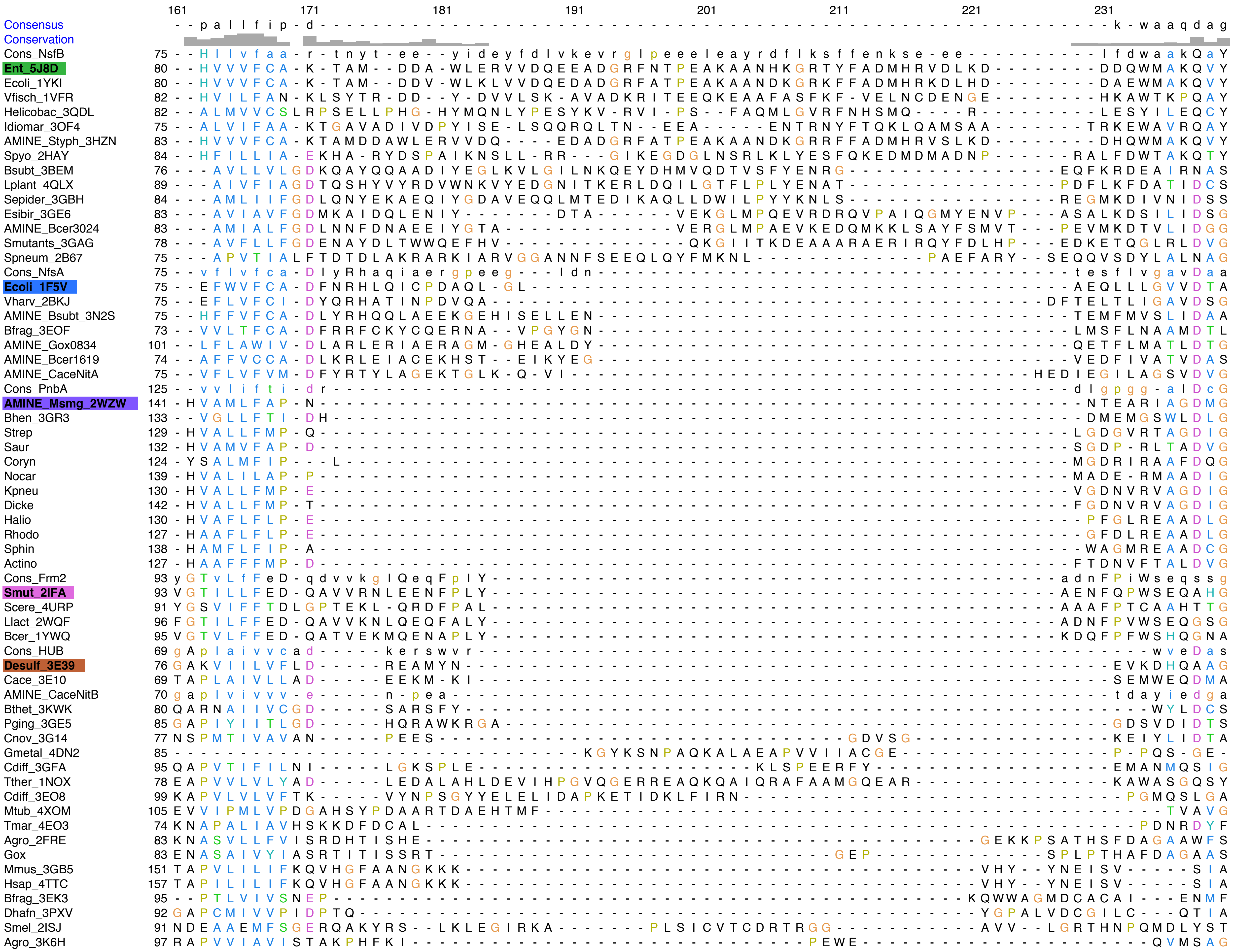
161
171
181
191
201
211
221
231
Consensus
Conservation
Cons_NsfB
Ent_5J8D
Ecoli_1YKI
Vfisch_1VFR
Helicobac_3QDL
Idiomar_3OF4
AMINE_Styph_3HZN
Spyo_2HAY
Bsubt_3BEM
Lplant_4QLX
Sepider_3GBH
Esibir_3GE6
AMINE_Bcer3024
Smutants_3GAG
Spneum_2B67
Cons_NfsA
Ecoli_1F5V
Vharv_2BKJ
AMINE_Bsubt_3N2S
Bfrag_3EOF
AMINE_Gox0834
AMINE_Bcer1619
AMINE_CaceNitA
Cons_PnbA
AMINE_Msmg_2WZW
Bhen_3GR3
Strep
Saur
Coryn
Nocar
Kpneu
Dicke
Halio
Rhodo
Sphin
Actino
Cons_Frm2
Smut_2IFA
Scere_4URP
Llact_2WQF
Bcer_1YWQ
Cons_HUB
Desulf_3E39
Cace_3E10
AMINE_CaceNitB
Bthet_3KWK
Pging_3GE5
Cnov_3G14
Gmetal_4DN2
Cdiff_3GFA
Tther_1NOX
Cdiff_3EO8
Mtub_4XOM
Tmar_4EO3
Agro_2FRE
Gox
Mmus_3GB5
Hsap_4TTC
Bfrag_3EK3
Dhafn_3PXV
Smel_2ISJ
Agro_3K6H

```
                     241       251       261       271       281       291       301       311
Consensus                laaqnlllaaaalglgscpiggfd---aevvre---gle----kllfgiafGy-pd-----ng-pkpr-p-leevvhf-
Conservation
Cons_NsfB            137 ialgnlltaaAelgiDscPmEGFd---pekvdeiL--gldekglrsvvmlalGYrd-ee-ddv-lpKvRkp-leevvtei
Ent_5J8D             145 LNVGNFLLGVGAMGLDAVPIEGFD---AAILDEEF--GLKEKGFTSLVVVPVGHHSVEDFN-ATLPKSRLP-LSTIVTEC
Ecoli_1YKI           145 LNVGNFLLGVAALGLDAVPIEGFD---AAILDAEF--GLKEKGYTSLVVVPVGHHSVEDFN-ATLPKSRLP-QNITLTEV
Vfisch_1VFR          145 LALGNALHTLARLNIDSTTMEGID---PELLSEIF--ADELKGYECHVALAIGYHHPSEDYNASLPKSRKA-FEDVITIL
Helicobac_3QDL       142 IAVGQICMGVSLMGLDSCIIGGFD---PLKVGEVL--EERINKPKIACLIALGK----RVAEASQ-KSRKS-KVDAITWL
Idiomar_3OF4         137 IGLGTFLLAAAELEVDSCPMEGIE---HDAYDNIL--SLKDLGLSTVFACPVGYRSEADTT-QFQKKVRQP-LSRFKVVL
AMINE_Styph_3HZN     148 LNVGNFLLGVAAMGLDAVPIEGFD---AEVLDAEF--GLKEKGYTSLVVVPVGHHSVEDFN-AGLPKSRLP-LETTLTEV
Spyo_2HAY            150 IALGNMMMTAALLGIDTCPIEGFH---YDKVNHILAKHNVIDLEKEGIASMLSL--GYRLRDPKHAQVRKP-KEEVMSVV
Bsubt_3BEM           137 LSAMMFMLSAAAAGWDTCPMIGFDAE----AVKR-I-LNIDDQFEVVMMITIGKEKTES---RRPRGYRKPVNEFVEYM-
Lplant_4QLX          150 VVGMQLLLVARAHGYDANAFSGIDFE----KMIP-TLGLDPKRYVPVMGIAIGK--AAQEPLHTTRYDAKTQTDFLA---
Sepider_3GBH         145 LMAMQLMLTAKAHGYDTNPIGGFDKE----NIAD-IIGYDSDRYVPVLAIAIGK--KAQ---DAHDSVRLPIDDVREFL-
Esibir_3GE6          144 LVSMQLMLVARAHGYDTNPIGGYEKD-----QIAEAFGMEKDRYVPVMLLSIGK--AVD---AGYPSVRLPINDIADWK-
AMINE_Bcer3024       144 LVAMQFMLAARAHGYDTCPIGGFEKD-----QIAEAFGLDKESYVPVMLISIGK--AAD---SGYQSVRLPIEKVAEWK-
Smutants_3GAG        138 LFAMNLMQVVRVYGYDSVPMRGVDFD------AIKTYLDMPNGWEPILMLPVGK----ALQ-AGNPHVRKSVAEFAEIIE
Spneum_2B67          137 LVAMNLVLALTDQGIGSNIILGFDKS--KVN--E-VLEIEDR-FRPELLITVGY-TDE-KLE-PSY--RLPVDEIIEKR-
Cons_NfsA            113 laaQnavvaAEslGLGivyiGgirn-npeevaelLgLPey---vfPvfGlcvGyPdq----epa-vKPRLP-leavlhee
Ecoli_1F5V           110 MMAQNALIAAESLGLGGVYIGGLRN-NIEAVTKLLKLPQH---VLPLFGLCLGWPAD----NPD-LKPRLP-ASILVHEN
Vharv_2BKJ           110 IMAQNCLLAAESMGLGGVYIGGLRNSAAQ-VDELLGLPEN---SAVLFGMCLGHPDQ----NPE-VKPRLP-AHVVVHEN
AMINE_Bsubt_3N2S     116 LAAQNMSIAAESMGLGICYIGGIRN-ELDKVTEVLQTPDH---VLPLFGLAVGHPANL----SG-KKPRLP-KQAVYHEN
Bfrag_3EOF           112 LVAQTFCTLAEEAGLGICYLGTTTY-NPQMIIDALHLPEL---VFPITTVTVGYPAE-----SPKQVDRLP-IEGIIHEE
AMINE_Gox0834        141 IAAQNAVTAFESLGLGTVYIGGLRN-HPEEVSSLLSLPPR---SVAVVGLCVGKPDPAA--QNG-YKPRLG-QDVVLHRE
AMINE_Bcer1619       113 LFAQNLALAAESLGYGICYIGGIRN-NPREVSELLHLPDK---VYPVFGMTVGVPDE----DHG-VKPRLP-VAAVLHEN
AMINE_CaceNitA       115 IALGASVVAAESLGLGTVPIGGIRK-NPEEVIEILGLPKY---TFPMVGLVVGYPAD----ESH-KKPRVP-FESFRHNE
Cons_PnbA            145 mflqnfmlaararGlgtcpQaalam--yspvvrehlgipddr--mivcgislGy-aDpdapaNsfrteRap-veefvtfv
AMINE_Msmg_2WZW      161 IYAQTLMLAMTAHGIASCPQALLSF--YADTVRAELGV-ENR--KLLMGISFGY-ADDTAAVNGVRIPRAG-LSETTRFS
Bhen_3GR3            153 MFMQTIMLAARGFGLDTCAQAAFAD--YHKQIRSLLSVPSDR--HIICGMALGY-RDMNAPENNFETEREP-IDNFVHFI
Strep                150 MYAQNFLLSLTARGFHGIPQAVLGV--YADTVRASLGVRDDL--KLLFGISFGI-GDKTSPMYDIGMGRIP-LQQSVVLH
Saur                 152 MYAQTLLLALNAYGVASCPQALMSF--FADTVRETLGLTGG---KLLFGIAFGY-ADPAAAVNTVAVGREP-LRETTRFH
Coryn                145 TYAENFLLSLTAHGYHGVPQGMISL--LAPTVREFLGVDEEY--KLVTALTFGR-ADESSPVYKTAPGRVA-LSETVTVH
Nocar                159 MYGQTLLLAMTAYGIASCPQGLLGF--YGETIRHQLGITDG---KVLFGVSFGY-ANTSAAVNRIRVARAK-LCETTRFH
Kpneu                151 MYAQTLLLSLTAHGLGGVPQTSLGF--YGETIRKELGVDPSL--KLLFGISLGY-PDITHPANQYRINKVS-LGESVTFH
Dicke                163 MYGQTFLLSLAAHGLGGIPQTSLGF--FADPIRHFLGIPDNL--KLLFGISFGY-PDEAAPGNRLRMDRVN-ISDSVTFH
Halio                150 MYAQTLMLAMAANGLGSCPQTALSF--QADLVREVLEIDASN--KLMFGISFGH-PDPDAPVNRCVTDRAL-LSDTVHFH
Rhodo                147 GYAQTLMLAMASRGIGSCAQGALSL--YPEIVRAHLGLVQGP--RLIMGIAFGY-KDPDHPANAAHTDRAA-LADIVHHH
Sphin                158 MYAQSFMLALTANGLASCAQGALSH--HAEIVHRELGVGEDQ--KLLFGIAFGY----EDAAHPANAARTV-RDKDHVLH
Actino               148 MLAQNFMLSLTAHGFGSVPQIALTY--SPDVVRQEFNVPDNY--KLVLGISFGR-PKPNSTANSYIQPRAP-LSETVVFH
Cons_Frm2            129 mlQfavWtaLeeeGlGAsLQHYNPIIDeaVake----wniPasWkLrAQmpFGsieap-p-geKefqp----leeRvkvf
Smut_2IFA            129 IALYAIWLALAEQNIGMSVQHYNPLVDAQVAEK----YDLPTNWKMRAQIPFGS-IEA-PAGEKEFMA----DQERFKVF
Scere_4URP           127 AVQIQSWTALELLGLGANLQHYNDYVKSALP-Q-----DVPIAWTVQSQLVFG---V--PTALPEEK----TFINNVINV
Llact_2WQF           132 IISVNVWTALAELGLGANLQHYNPLIDEAVAKE----WNLPESWKLRGQLVFGS-IEA-PAGEKTFMD----DADRFIVA
Bcer_1YWQ            131 MLQHTVWMLLSAEGIGASLQHYNPIVDAEVKET----WNIPAEWSLVGQMPFGE-PNEQPA-ERTFLP----TEDVVKFY
Cons_HUB              93 iaaenmlLaAtelGLGscwvgif--de--ekvrell-glP-enlepvaliplGy-pa---eepkppdrk-p-leeivyn-
Desulf_3E39          102 AAVQNMLLAAHALQLGAVWL--GEIINQAATLLPAL-ALDPARLSFEAAIAAGH-PA---QNGSSS-RR-P-LAELLLEE
Cace_3E10             95 AAAENILLEAAYLDLGAVWLGAQPIEERVKNLKEMF-NLK-SNIKPFCVISVGY-PE--NSENKFIDRF-D-AKRIHIEK
AMINE_CaceNitB        93 iastliqvtahsmglgscw--------chvrnrdri-dydtt--eafiktllni-penlkvecmlgigy-pdeerkaytd
Bthet_3KWK           103 AAAQNILLAAESMGLGAVWTAAYPYEDRMEVVRKYT-HLPEN-ILPLCVIPFGY-PA---TKEQPKQKY-D-EKKIHYNQ
Pging_3GE5           114 IAMTYMMLEAHSLGLGCTWVCAF----DQALCSEIF-DIPSH-MTPVSILALGY-GDPTVPPREAFNRK-T-IEEVVSFE
Cnov_3G14            106 IAMEHIVLGATDEGYGTCWIAAF----NENKIKEAL-KIPDN-LRVVALTPLGV-PKDSAEDEPHHPKK-D-MDEYLYID
Gmetal_4DN2          115 LRGQQYYLT--DVGIAAQNLMLAAHDLGLG---SVFVGVF-DE--QQLGELLGI-PAE--LRIVGLFPL-G-YPL--EGP
Cdiff_3GFA           131 AAIQNMSLTAVELGLGSLWICDV-Y-FAYRELCEWL-NT--D-SQLVAAISLGY-PD---EEPSRRPRL-Q-LSDVTEWR
Tther_1NOX           138 ILLGYLLLLLEAYGLGSVPM----LGFDPERVRAIL-GLPSR-AAIPALVALGY-PA---EEGYPSHRL-P-LERVVLWR
Cdiff_3EO8           145 AIENFTLSAIELGYGSCWLTSQNYAADEIEAVLEAETGFEKGEYFLGAMLALGV-PE---DNLKSPSKK-P-VEEICTFI
Mtub_4XOM            138 AAVQALLVALAVRGLGSCWIGSTIFAADLVRDELDLPV----DWEPLGAIAIGYADEPSGL--RDPVPAA----DLLILK
Tmar_4EO3            100 LFDTGLAV-----GNLLVQATQM--------------------GLVAHPVAGYDPV--KVKE-I--LKIPED-HVLI-T
Agro_2FRE            121 LAMQAHLLGYHAHGMGGIFKD---------------RIVEKLDIPDGFKVEAGVAIG--TLTDKS---ILPDD--LAERE
Gox                  120 VLIQI---QASQAGWAVHPISG------FDRDLAHAGLEFPEDYQVHAALVIGRQGDAGALSEELREREQPSG-----RF
Mmus_3GB5            185 CGLLLAALQNAGLVTVTTTPLNCGP---RLRV-L---LGRPSHEKLLVLLPVGY-PSR------DA-TVPDLKRKAL---
Hsap_4TTC            191 CGILLAALQNAGLVTVTTTPLNCGP---RLRV-L---LGRPAHEKLLMLLPVGY-PSK------EA-TVPDLKRKPL---
Bfrag_3EK3           122 LAA-----TSLGIASCWINQLGTTCDDPEVRAYLTS-LGVPENHKVYGCVALGY-----------K------AE-GALLKE
Dhafn_3PXV           122 LAA-----TSLGIANIMCGYTGLA-FASGLRA---EEFS----KRL-GF-PEGYAFGCSVLL-GH----------------
Smel_2ISJ            149 VCAVQNLWLAARAEGVGVGWV----SIFHESEIKAIL--GIPDHVEIVAWLCLGF---VD----RLYQEPELAAKGWRQR
Agro_3K6H            125 AVCLNVIFAANASGFAANWLTEWLAFDPA----FLAEIGV----SAE-EKVAGYIHIGS------------TTF-PPVERPR
```

		321 331 341 351 361 371 381 391
Consensus		---
Conservation		
Cons_NsfB	207	---
**Ent_5J8D**	217	---
Ecoli_1YKI	217	---
Vfisch_1VFR	218	---
Helicobac_3QDL	210	---
Idiomar_3OF4	209	---
AMINE_Styph_3HZN	220	---
Spyo_2HAY	224	K--
Bsubt_3BEM	206	---
Lplant_4QLX	219	---
Sepider_3GBH	213	---
Esibir_3GE6	212	---
AMINE_Bcer3024	212	---
Smutants_3GAG	206	---
Spneum_2B67	204	---
Cons_NfsA	183	tY---tde----ealaaYdetlseyyqertsnqr--ttwseqiaarlskegr----phlleflkkqGfllr
**Ecoli_1F5V**	180	SYQPLD-----KGALAQYDEQLAEYYLTRGSNNRR-DTWSDHIRRTIIKESR----PFILDYLHKQGWATR
Vharv_2BKJ	180	QYQELN-----LDDIQSY-QTMQAYYASRTSNQKL-STWSQEVTGKLAGESRSK--GLAKR----------
AMINE_Bsubt_3N2S	186	TYNVNTDD-F-RHTMNTYDKTISDYYRERTNGKRE-ETWSDQILNFMKQKPR----TYLNDYVKEKGFNKN
Bfrag_3EOF	182	SYHDYTAEDINRLYAYKESLPENKLFIEENQKETLPQVFT-DVRYTKKDN-EFMSENLLK-VLRRQGFMD-
AMINE_Gox0834	213	RY--STENEA-KGIAAY-DRIADDYQKEQGLTSRV---WSETVAKRVDSFKGLSGRHVIRTVLHRLGFPLR
AMINE_Bcer1619	183	GYDEQKYDE----LLNEYDETMNAYYKERSSNKKN-VTWTESMSSFMSKE-KR---MHMKEFLSEKGLNKK
AMINE_CaceNitA	185	SYDIKAVED----SINVYEQ-MNKYLKEIGRAEKE-INW-STFTSTIYQ------SVYYKQGLKTK-----
Cons_PnbA	219	g--
**AMINE_Msmg_2WZW**	234	R--
Bhen_3GR3	227	KSYP---
Strep	224	DTPGVLDEQ--
Saur	225	R--
Coryn	219	GIEGLGL--
Nocar	232	T--
Kpneu	225	Q--
Dicke	237	N--
Halio	224	D--
Rhodo	221	GDT--
Sphin	229	G--
Actino	222	E--
Cons_Frm2	199	k--
**Smut_2IFA**	199	GDLE---
Scere_4URP	192	YH---
Llact_2WQF	202	K--
Bcer_1YWQ	200	---
Cons_HUB	159	---
**Desulf_3E39**	172	PFPQPE---
Cace_3E10	168	Y--
AMINE_CaceNitB	160	edlalnklhyd-kysk---
Bthet_3KWK	175	Y--
Pging_3GE5	185	KL---
Cnov_3G14	177	KWGTSFMESNVKILEKN--
Gmetal_4DN2	180	KAGPSRKPLDEIVHYGKYQA---
Cdiff_3GFA	198	---
Tther_1NOX	205	---
Cdiff_3EO8	219	K--
Mtub_4XOM	207	---
Tmar_4EO3	147	LIAVGYLGDESELSEKHRELERSERVRKELSEIVRWNL---------------------------------
Agro_2FRE	179	VPSKRVPLADVAFEGRFTGKAD---
Gox	186	PLA-EIAFEGRFRSNE---
Mmus_3GB5	247	-DQIMVTV---
Hsap_4TTC	253	-DQIMVTV---
Bfrag_3EK3	179	KTVK----AGTITIVE---
Dhafn_3PXV	171	---A-NTTKPPHVPD----KDKITYVE--
Smel_2ISJ	216	L-----------PLEDLVFEEGWGVR---
Agro_3K6H	185	P-----------ELADVVTWVGDV---

4. References

1. Phillips, K. L.; Sandler, S. I.; Chiu, P. C., A method to calculate the one-electron reduction potentials for nitroaromatic compounds based on gas-phase quantum mechanics. *J. Comput. Chem.* **2011,** 32, (2), 226-239.
2. Salter-Blanc, A. J.; Bylaska, E. J.; Johnston, H. J.; Tratnyek, P. G., Predicting reduction rates of energetic nitroaromatic compounds using calculated one-electron reduction potentials. *Environ. Sci. Technol.* **2015,** 49, 3778-3786.
3. La-Scalea, M. A.; Menezes, C. M. S.; Juliao, M. S. S.; Chung, M. C.; Serrano, S. H. P.; Ferreira, E. I., Voltammetric Behavior of Nitrofurazone and its Hydroxymethyl Prodrug with Potential Anti-Chagas Activity. *J. Braz. Chem. Soc.* **2005,** 16, (4), 774-782.
4. Hansch, C.; Leo, A.; Taft, R. W., A survey of Hammett substituent constants and resonance and field parameters. *Chem. Rev.* **1991,** 91, (2), 165–195.
5. Pettersen, E. F.; Goddard, T. D.; Huang, C. C.; Couch, G. S.; Greenblatt, D. M.; Meng, E. C.; Ferrin, T. E., UCSF Chimera - a visualization system for exploratory research and analysis. *J. Comput. Chem.* **2004,** 25, (13), 1605-1612.
6. Gwenin, V. V.; Poornima, P.; Halliwell, J.; Ball, P.; Robinson, G.; Gwenin, C. D., Identification of novel nitroreductases from *Bacillus cereus* and their interaction with th CB1954 prodrug. *Biochem. Pharmacol.* **2015,** 98, 392-402.
7. Chaignon, P.; Cortial, S.; Ventura, A. P.; Lopes, P.; Halgand, F.; Laprevote, O.; Ouazzani, J., Purification and identification of a Bacillus nitroreductase: Potential use in 3,5-DNBTF biosensoring system. *Enz. Microb. Technol.* **2006,** 39, 1499-1506.
8. Yang, Y.; Lin, J.; Wei, D., Heterologous overexpression and biochemical characterization of a nitroreductase from Gluconobacter oxydans 621H. *Mol. Biotechnol.* **2016,** 58, 428-440.
9. Kutty, R.; Bennett, G. N., Biochemical characterization of trinitrotoluene transforming oxygen-insensitive nitroreductases from Clostridium acetobutylicum ATCC 824. *Arch Microbiol* **2005,** 184, 158-167.
10. Manina, G.; Bellinzoni, M.; Pasca, M. R.; Neres, J.; Milano, A.; Ribeiro, A. L. J. L.; Buroni, S.; Skovierova, H.; Dianiskova, P.; Mikusova, K.; Marak, J.; Makarov, V.; Giganti, D.; Haouz, A.; Lucarelli, A. P.; Degliacomi, G.; Piazza, A.; Chiarelli, L. R.; De Rossi, E.; Salina, E.; Cole, S. T.; Alzari, P. M.; G., R., Biological and structural characterization of the *Mycobacterium smegmatis* nitroreductase NfnB, and its role in benzothiozinone resistance. *Mol. Microbiol.* **2010,** 77, (5), 1172-1185.
11. Akiva, E.; Copp, J. N.; Tokuriki, N.; Babbitt, P. C., Evolutionary and molecular foundations of multiple contemporary functions of the nitroreductase superfamily. *Proc Natl Acad Sci U S A* **2017,** 114, (45), E9549–E9558.
12. Akiva, E.; Brown, S.; Almonacid, D. E.; Barber, A. E.; Custer, A. F.; Hicks, M. A.; Huang, C. C.; Lauck, F.; Mashiyama, S. T.; Meng, E. C.; Mischel, D.; Morris, J. H.; Ojha, S.; Schnoes, A. M.; Stryke, D.; Yunes, J. M.; Ferrin, T. E.; Holliday, G. L.;

Babbitt, P. C., The Structure-Function Linkage Database. *Nucl. Acids Res.* **2014,** 42, D521-D530.

13. Zubatyuk, R. I.; Gorb, L.; Shishkin, O. V.; Qasim, M.; Leszczynski, J., Exploration of density functional methods for one-electron reduction potential of nitrobenzenes. *J. Comput. Chem.* **2-1-,** 31, 144-150.

© 2017 by the authors. Submitted for possible open access publication under the terms and conditions of the Creative Commons Attribution (CC-BY) license (http://creativecommons.org/licenses/by/4.0/).
